# DNA‐PKcs‐Driven YAP1 Phosphorylation and Nuclear Translocation: a Key Regulator of Ferroptosis in Hyperglycemia‐Induced Cardiac Dysfunction in Type 1 Diabetes

**DOI:** 10.1002/advs.202412698

**Published:** 2025-04-25

**Authors:** Junyan Wang, Xing Chang, Chun Li, Jing Gao, Zhijiang Guo, Haowen Zhuang, Lingjun Wang, Yusheng Huang, Wei Wang, Chao Li, Qingyong He

**Affiliations:** ^1^ State Key Laboratory of Traditional Chinese Medicine Syndrome, School of Pharmaceutical Sciences Guangzhou University of Chinese Medicine Guangzhou Guangdong 510006 China; ^2^ Guang'anmen Hospital China Academy of Chinese Medical Sciences Beijing 100053 China; ^3^ The First Affiliated Hospital Guangzhou University of Chinese Medicine Guangzhou 510405 China; ^4^ College of Traditional Chinese Medicine Shandong University of Traditional Chinese Medicine Jinan 250355 China

**Keywords:** diabetic cardiomyopathy, DNA damage response, DNA‐PKcs, ferroptosis, YAP1

## Abstract

The DNA‐Dependent Protein Kinase catalytic subunit (DNA‐PKcs) acts as a principal executor in the DNA damage response (DDR), mediating the phosphorylation of a broad spectrum of substrates integral to DNA repair and apoptosis. This investigation seeks to discern the possible association and mechanisms linking hyperglycemia‐induced ferroptosis and DNA‐PKcs in DCM. This data exhibits a substantial activation of DNAPKcs‐ dependent DDR in mice with streptozotocin‐induced DCM. However, deletion of *DNA‐PKcs* in cardiomyocytes notably mitigates DNA damage, enhances heart function and dampens the inflammatory response. Co‐IP/MS analysis and subsequent validation experiments demonstrate that *DNA‐PKcs* directly interacts with and phosphorylates YAP1 at Thr226. This phosphorylation event facilitates the nuclear retention of YAP1, where it intensifies the transcription of ferroptosis‐associated genes. Knockin mice expressing a nonphosphorylatable T226A YAP1 mutant display decreased ferroptosis, reduced myocardial fibrosis and improved heart function. Taken together, this study unravels that DDR acts as an intracellular stress damage sensor, perceiving hyperglycemic conditions and subsequently transmitting the damage signal to incite ferroptosis through the interplay between DNA‐PKcs and YAP1. This novel insight suggests that the DNA‐PKcs‐mediated YAP1 phosphorylation and the ferroptosis activation could be the promising therapeutic targets for the management of DCM.

## Graphical abstract

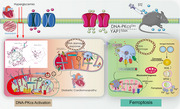

## 1 Introduction

Diabetic cardiomyopathy (DCM) is a critical and distinct complication of diabetes, characterized by structural and functional impairments of the heart, independent of traditional cardiovascular risk factors like hypertension and coronary artery disease^[^ ^1^ ^]^ DCM is a leading contributor to heart failure‐related morbidity and mortality among diabetic patients, exacerbating the global burden of cardiovascular disease. The underlying pathogenesis of DCM is multifactorial, driven primarily by hyperglycemia‐induced metabolic derangements such as mitochondrial dysfunction, oxidative stress, and the accumulation of advanced glycation end products (AGEs), which collectively disrupt myocardial homeostasis.^[^ ^2^ ^]^ Recent evidence has highlighted the pivotal role of the DNA damage response (DDR) in metabolic disorders, including diabetes.^[^ ^3^ ^]^ The DDR is activated by a variety of stressors, such as oxidative damage and telomere shortening, which are prevalent in the diabetic milieu.^[^ ^4^ ^]^ At the core of DDR is the DNA‐dependent protein kinase catalytic subunit (DNA‐PKcs), which coordinates DNA repair, cell cycle regulation, and apoptosis through the phosphorylation of numerous substrates.^[^ ^5^ ^]^ While DNA‐PKcs has been linked to the development of diabetic kidney disease^[^ ^6^ ^]^ and pancreatic islet inflammation,^[^ ^7^ ^]^ its specific role in DCM remains unexplored.

DDR, traditionally regarded as a protective mechanism for maintaining genomic stability,^[^ ^8^ ^]^ can paradoxically lead to cell death under conditions of sustained or excessive DNA damage.^[^ ^9^ ^]^ In metabolic diseases like diabetes, chronic DDR activation may promote cellular senescence and cardiomyocyte dysfunction, implicating DDR as a key driver of DCM.^[^ ^10^ ^]^ Our previous studies have demonstrated that DNA‐PKcs contributes to cellular apoptosis in septic cardiomyopathy^[^ ^11^ ^]^ and ischemia‐reperfusion injury,^[^ ^12^ ^]^ suggesting a similar role in DCM.

Ferroptosis, a form of regulated cell death driven by iron‐dependent lipid peroxidation, has emerged as a novel pathological mechanism in DCM.^[^ ^13^ ^]^ Hyperglycemia and insulin resistance, hallmarks of diabetes, are known to induce oxidative stress and inflammation, creating an environment conducive to ferroptosis.^[^ ^14^ ^]^ This form of regulated cell death is characterized by excessive lipid peroxidation and the accumulation of reactive oxygen species, which contribute significantly to cardiac dysfunction in DCM.^[^ ^15^ ^]^ Chronic hyperglycemia and dysregulated lipid metabolism in diabetes foster mitochondrial oxidative stress and iron accumulation, thereby sensitizing cardiomyocytes to ferroptotic damage.^[^ ^16^ ^]^ Impaired Glutathione Peroxidase 4 (GPX4) function and reduced glutathione levels further increase susceptibility to ferroptosis, while proinflammatory signaling pathways, such as Nuclear Factor kappa‐light‐chain‐enhancer of activated B (NF‐κB) and Nuclear Factor Erythroid 2‐Related Factor 2 (Nrf2), exacerbate the resultant damage.^[^ ^17^ ^]^ Potential therapeutic strategies, including iron chelation, GPX4 activation, and antioxidant treatments, show promise in mitigating ferroptosis‐associated cellular injury and preserving cardiac function in diabetic conditions.^[^ ^18^ ^]^ However, the role of DNA‐PKcs activation, mediated by the DDR, in contributing to ferroptosis in DCM remains to be fully elucidated.

Yes‐associated protein (YAP), a central effector of the Hippo signaling pathway, has been implicated in the regulation of ferroptosis.^[^ ^19^ ^]^ YAP has been shown to modulate redox homeostasis and iron metabolism, key factors in ferroptosis, through the regulation of antioxidant^[^ ^20^ ^]^ and iron metabolism‐related genes.^[^ ^21^ ^]^ Additionally, YAP interacts with p53, a well‐known mediator of DDR^[^ ^22^ ^]^ and ferroptosis.^[^ ^23^ ^]^ Given this, we hypothesize that hyperglycemia‐induced DDR may trigger ferroptosis in DCM via a YAP‐dependent mechanism. This study aims to investigate whether hyperglycemia activates DDR through DNA‐PKcs, leading to YAP‐mediated ferroptosis in cardiomyocytes. By elucidating the molecular interplay between DNA‐PKcs and YAP in the context of ferroptosis, we hope to identify novel therapeutic targets for the management of DCM.

## 2 Results

### 2.1 DNA Damage Response is Activated in DCM

To explore potential links between high‐glucose stress and transcriptional changes in diabetic cardiomyopathy (DCM), we conducted bioinformatics analyses using publicly available RNA‐seq data from the GSE106180 dataset, which includes heart tissues from streptozotocin (STZ)‐induced diabetic mice (Figure S2A,B, Supporting Information). KEGG and GO analyses demonstrated significant involvement of genes associated with “double‐strand break repair,” “cell cycle G2/M transition,” and “DNA damage checkpoint signaling” in diabetic hearts (Figure S2C–F, Supporting Information). GSEA also confirmed increased double‐strand break repair, DNA damage response, nonhomologous end joining, and DNA repair, in diabetic hearts (Figure S2G, Supporting Information).

**Figure 1:**
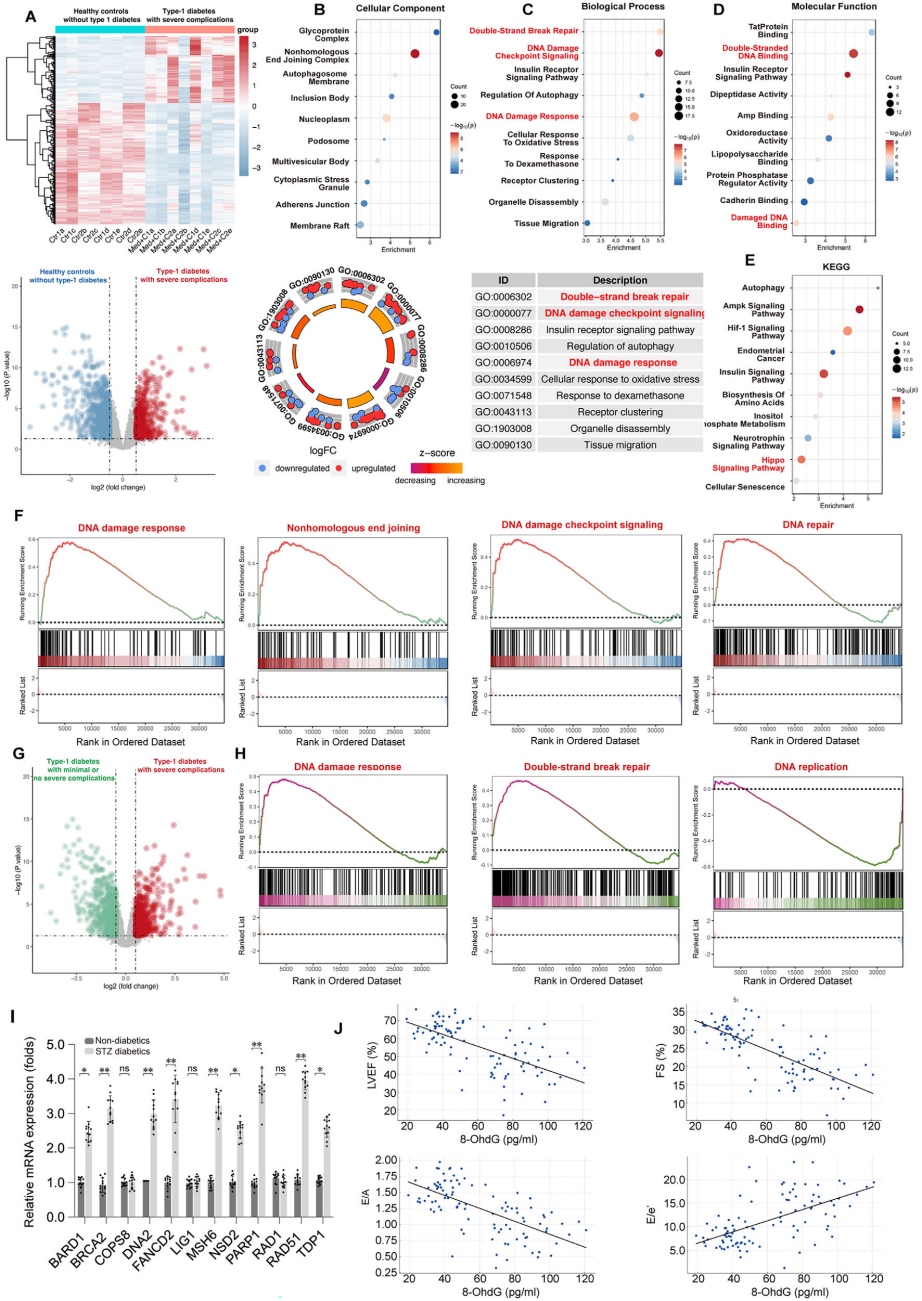
Activation of the DDR in T1D DCM. A) Bioinformatics analysis performed on publicly available GSE70752 RNA‐seq data from primary human fibroblast samples from six healthy controls without type 1 diabetes (T1D), six patients with longstanding (≥50 years) T1D who have minimal to no severe complications (designated Medalist‐C, Med‐C), and six patients with longstanding (≥50 years) T1D who have severe complications (designated Medalist+C, Med+C). B–D) Gene Ontology (GO) Biological Process, Cellular Component, and Molecular Function analysis showing the enrichment of genes associated with DDR activation. E) Kyoto Encyclopedia of Genes and Genomes (KEGG) pathway analysis of differentially expressed genes between healthy controls and T1D patients with severe complications. F) Gene Set Enrichment Analysis (GSEA) plots illustrating significant enrichment of DDR‐related processes. G) The distribution of differentially expressed genes between T1D patients with minimal to no severe complications and T1D patients with severe complications. H) Gene Set Enrichment Analysis (GSEA) plots illustrating significant enrichment of DDR‐related processes. I) Quantitative PCR (qPCR) validation of selected DDR‐related genes. Each group consisted of 4 animals (*n* = 4). For each animal, measurements were repeated three times under the same experimental conditions. In this panel, dots represent individual measurements from animals. Bars represent group means, and error bars indicate ± standard error (SD). *p < 0.05, **p < 0.01, ***p < 0.001, ns, not significant. J) Correlation analysis between oxidative DNA damage marker 8‐hydroxy‐2′‐deoxyguanosine (8‐OHdG) levels and cardiac function parameters, including left ventricular ejection fraction (LVEF), fractional shortening (FS), E/A ratio, and E/e’ ratio. *n* = 50 patients per group and dots in this panel represent the average data of three replicates in each patient.

To elucidate the molecular mechanisms underlying diabetic cardiomyopathy and its associated severe complications in type 1 diabetes (T1D), we analyzed transcriptomic data from the GSE70752 dataset. This dataset includes primary human fibroblast samples from six healthy controls (non‐T1D), six individuals with longstanding (≥50 years) T1D who exhibit minimal or no severe complications (designated Medalist‐C, Med‐C), and six individuals with longstanding T1D who present with severe complications (designated Medalist+C, Med+C). Differentially expressed gene (DEG) analysis revealed distinct expression profiles between the healthy controls (Control group) and T1D patients with severe complications (Med+C group) (**Figure** **1**A). Gene Ontology (GO) Biological Process enrichment analysis demonstrated significant alterations in pathways related to “Double‐Strand Break Repair,” “DNA Damage Checkpoint Signaling,” and the broader “DNA Damage Response” (Figure 1B–D). GO Molecular Function analysis further highlighted the predominance of terms such as “Double‐Stranded DNA Binding” and “Damaged DNA Binding” in T1D patients with severe complications (Figure 1B–D). Concurrently, Kyoto Encyclopedia of Genes and Genomes (KEGG) pathway enrichment analysis identified significant upregulation of the Hippo signaling pathway in T1D patients (Figure 1E).

To further dissect the dynamics of DNA damage response (DDR)‐related pathways, we performed Gene Set Enrichment Analysis (GSEA) to evaluate differences in pathway activity between the Med+C group and healthy controls. GSEA revealed significant activation of DDR‐related pathways, including “DNA Damage Response,” “Non‐Homologous End Joining,” “DNA Damage Checkpoint Signaling,” and “DNA Repair” (Figure 1F), underscoring heightened DDR pathway activation in T1D patients with severe complications.

To validate the pivotal role of DDR activation in the progression of severe T1D complications, we compared transcriptomic profiles between the Med+C and Med‐C groups. GSEA analysis of ranked gene expression confirmed persistent and significant upregulation of DDR‐related pathways in the Med+C group relative to the Med‐C group (Figure 1H). These findings collectively underscore the critical role of aberrant DDR pathway activation in driving the development and progression of severe complications in longstanding T1D (Figure 1G,H).

Next, to investigate the impact of glucose levels on DDR activation, we selected representative genes (*BARD1, BRCA2, COPS8*, *DNA2*, *FANCD2*, *LIG1, MSH6, NSD2, PARP1, RAD1, RAD51*, and *TDP1*) identified as differentially expressed in the GSE106180 dataset. STZ injection was used to establish a mouse model of DCM, and qPCR analysis confirmed upregulation of several DDR‐related genes, including *RAD51, BRCA2, DNA2, FANCD2, MSH6, TDP1, NSD2, PARP1*, and *BARD1*, in diabetic mice compared to controls (Figure 1I). However, *RAD1*, *LIG1*, and *COPS8* levels showed no significant difference between the groups, despite their differential expression in the original RNA‐seq data.

To further validate our bioinformatics data, we measured oxidative DNA damage in human samples by analyzing plasma 8‐hydroxydeoxyguanosine (8‐OHdG) levels, a marker of oxidative DNA damage. Plasma 8‐OHdG was significantly elevated in diabetic patients (*n* = 50) compared to non‐diabetic controls (*n* = 50), with higher levels correlating negatively with cardiac function, including left ventricular ejection fraction (LVEF), fractional shortening (FS), and diastolic function parameters (E/A and E/e’) (Figure 1J). Collectively, these findings corroborate the activation of DDR in DCM.

### 2.2 DDR is Controlled by DNA‐PKcs and Contributes to Hyperglycemia‐Mediated Cardiomyocyte Dysfunction

**Figure 2:**
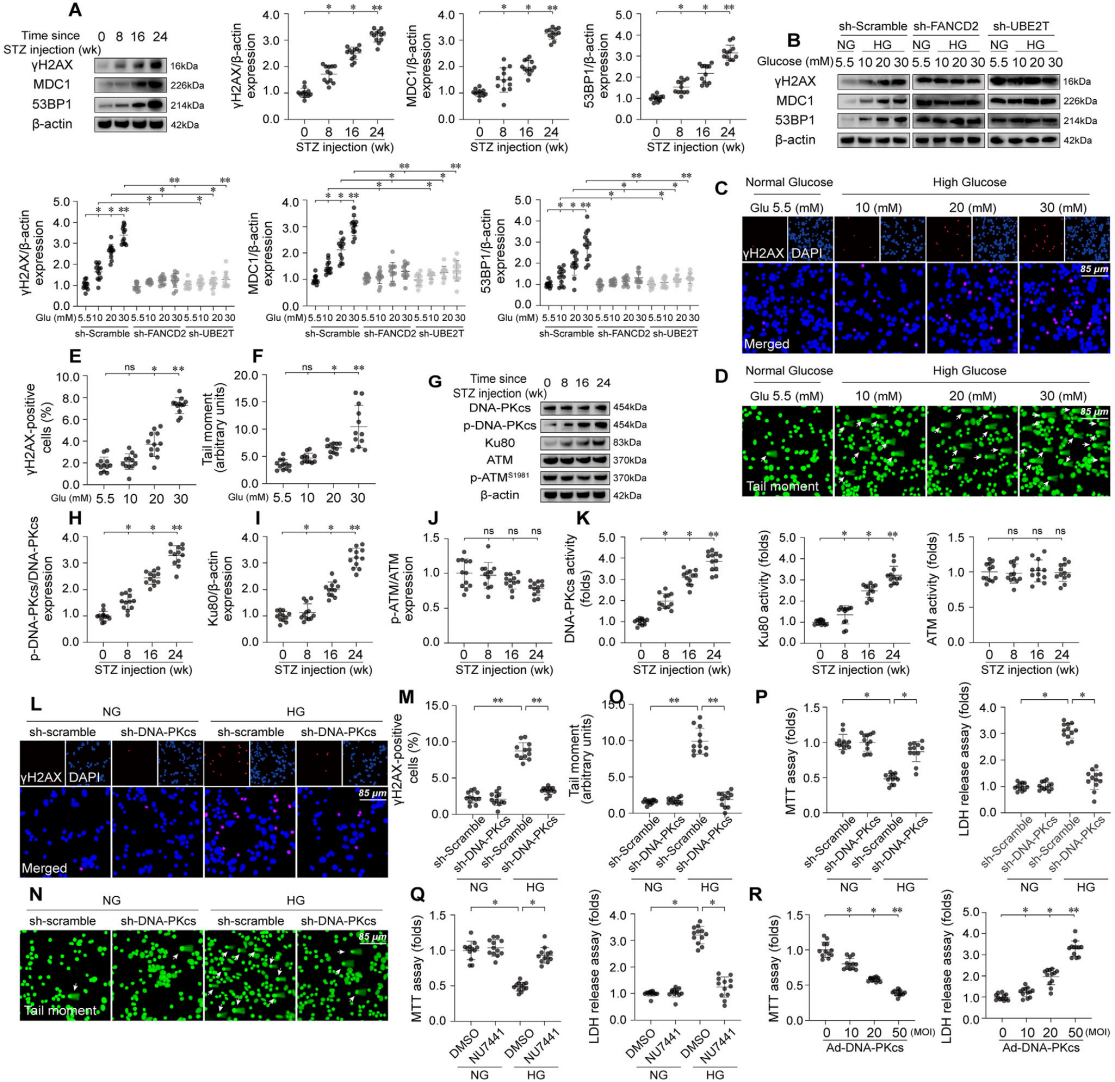
DNA‐PKcs regulates the DDR and contributes to hyperglycemia‐induced myocardial dysfunction. In vivo, cardiomyocyte‐specific *DNA‐PKcs* knockout (*DNA‐PKcs^Cko^*) and wild‐type *DNA‐PKcs^f/f^* mice were injected intraperitoneally with streptozotocin (STZ, 50 mg kg^−1^ in 0.1 mol L^−1^ citrate buffer) for five consecutive days to induce diabetes. Age‐ and sex‐matched non‐diabetic control mice were injected with PBS. In vitro, HL‐1 cardiomyocytes were cultured in high‐glucose (HG, 30 mmol L^−1^) medium for 48 h to mimic hyperglycemic stress, while cells incubated in normal glucose (NG, 5.5 mmol L^−1^) medium served as controls. A) Western blot analysis of DDR markers γH2AX, MDC1, and 53BP1 in protein extracts from STZ‐ or PBS‐treated mice. B) Western blot analysis of γH2AX, MDC1, and 53BP1 in HL‐1 cells transduced with sh‐scramble, sh‐FANCD2, or sh‐UBE2T, and exposed to HG or NG conditions. C) Immunofluorescence staining for γH2AX in HL‐1 cells exposed to HG stress. D) DNA damage assessment via comet assays in HG‐treated HL‐1 cells; arrows indicate nuclei with DNA damage. E) Quantification of γH2AX‐positive cells in response to HG stress. F) Quantification of comet assays in HL‐1 cells. G–J) Western blot analysis of phosphorylated DNA‐PKcs (p‐DNA‐PKcs), Ku80, and phosphorylated ATM (p‐ATM) in protein extracts from STZ‐ or PBS‐treated mice. K) ELISA for DNA‐PKcs, Ku80, and ATM activities in diabetic and non‐diabetic heart tissue. L) Immunofluorescence staining for γH2AX in HL‐1 cells transduced with sh‐scramble or sh‐DNA‐PKcs. M) Quantification of γH2AX‐positive cells in response to HG stress. N) Comet assays in HL‐1 cells transduced with sh‐scramble or sh‐DNA‐PKcs; arrows indicate nuclei with DNA damage. O) Quantification of comet assays in HL‐1 cells. P) MTT assay to evaluate cell viability in HL‐1 cells infected with sh‐DNA‐PKcs. ELISA to measure lactate dehydrogenase (LDH) levels in culture media from HL‐1 cells infected with sh‐DNA‐PKcs. Q) MTT assay to evaluate cell viability in HL‐1 cells treated with the DNA‐PKcs inhibitor NU7441. ELISA to measure LDH levels in culture media from HL‐1 cells treated with NU7441. R) MTT assay to assess cell viability in HL‐1 cells transduced with Ad‐DNA‐PKcs under HG conditions (30 mmol L^−1^). ELISA to measure LDH levels in culture media from HL‐1 cells transduced with Ad‐DNA‐PKcs under HG conditions (30 mmol L^−1^). Each group consisted of 4 animals or 4 independent cell culture experiments (*n* = 4). For each animal or independent cell culture experiment, measurements were repeated three times under the same experimental conditions. In each panel, dots represent individual measurements from animals or independent cell culture experiments. Bars represent group means, and error bars indicate ± standard error (SD). *p < 0.05, **p < 0.01, ***p < 0.001, ns, not significant.

To verify the effects of hyperglycemia on DDR in the heart, we conducted in vivo analysis by isolating heart tissues from diabetic mice. Western blot assays revealed a time‐dependent increase in the expression of DDR markers, such as γH2AX, MDC1, and 53BP1, in diabetic hearts compared to non‐diabetic controls (**Figure** **2**A). Similarly, HL‐1 cells treated with high glucose (HG) displayed a dose‐dependent elevation in the levels of γH2AX, MDC1, and 53BP1 relative to baseline conditions (Figure 2B). Immunofluorescence staining for γH2AX (Figure 2C,E) and comet assays (Figure 2D,F) further confirmed that hyperglycemia promotes DDR in HG‐treated HL‐1 cells.

To assess the direct relationship between DDR and cardiomyocyte dysfunction, short hairpin RNAs (shRNAs) targeting *FANCD2* and *UBE2T*, key components of DDR, were employed. Knockdown of either *FANCD2* or *UBE2T* resulted in a marked reduction in DNA damage (Figure 2B) and significantly improved cardiomyocyte viability, as evaluated by MTT assays (Figure S3A, Supporting Information and lactate dehydrogenase (LDH) release (Figure S3B, Supporting Information). Conversely, overexpression of UBE2T or FANCD2 led to a dose‐dependent increase in DDR activation (Figure S3C–H, Supporting Information) and cardiomyocyte dysfunction (Figure S3I,J, Supporting Information) under both normal and high‐glucose (HG) conditions.

DDR is primarily regulated by three key proteins: the Ku70/Ku80 heterodimer, DNA‐PKcs, and ATM, with Ku70/Ku80 and DNA‐PKcs being the primary sensors of double‐strand breaks. We investigated the roles of DNA‐PKcs, Ku80, and ATM in hyperglycemia‐induced DDR within the heart. Western blot analysis revealed that phosphorylation of DNA‐PKcs and Ku80 was significantly elevated in diabetic hearts in a dose‐dependent manner, while ATM phosphorylation showed no significant change in response to hyperglycemia (Figure 2G–J). ELISA results confirmed the upregulation of DNA‐PKcs and Ku80 activity, but not ATM, in the diabetic heart (Figure 2K).

γH2AX immunofluorescence (Figure 2L,M) and comet assays (Figure 2N,O) demonstrated that *DNA‐PKcs* knockdown significantly attenuated HG‐induced DDR, which was followed by an improvement in cell viability (Figure 2P). However, knockout of *Ku80* had no cardioprotective effects on HG‐treated HL‐1 cells (Figure S3K, Supporting Information). Consistent with these findings, treatment with the DNA‐PKcs inhibitor NU7441 modestly enhanced cell viability under HG stress (Figure 2Q), whereas the Ku80 inhibitor STL127705 had no discernible effect on cardiomyocyte function (Figure S3L, Supporting Information). Interestingly, DNA‐PKcs overexpression correlated with reduced cardiomyocyte viability under both physiological and hyperglycemic conditions, in contrast to Ku80 (Figure 2R and Figure S3M, Supporting Information). These results suggest that DDR, modulated by DNA‐PKcs, is activated by hyperglycemia and plays a significant role in contributing to myocardial injury in diabetic cardiomyopathy.

### 2.3 Deletion of *DNA‐PKcs* Mitigates Hyperglycemia‐Induced Cardiac Dysfunction

To determine if aberrant DNA‐PKcs expression contributes to DCM, we engineered a cardiomyocyte‐specific *DNA‐PKcs* knockout mouse model (*DNA‐PKcs^Cko^*). In parallel, to evaluate whether hyperglycemia‐induced heart dysfunction was solely dependent on DNA‐PKcs, we developed a cardiomyocyte‐specific *Ku80* knockout (*Ku80^Cko^*) mouse model. Knockdown was confirmed by Western blot analyses (Figure S4A,B, Supporting Information).

**Figure 3:**
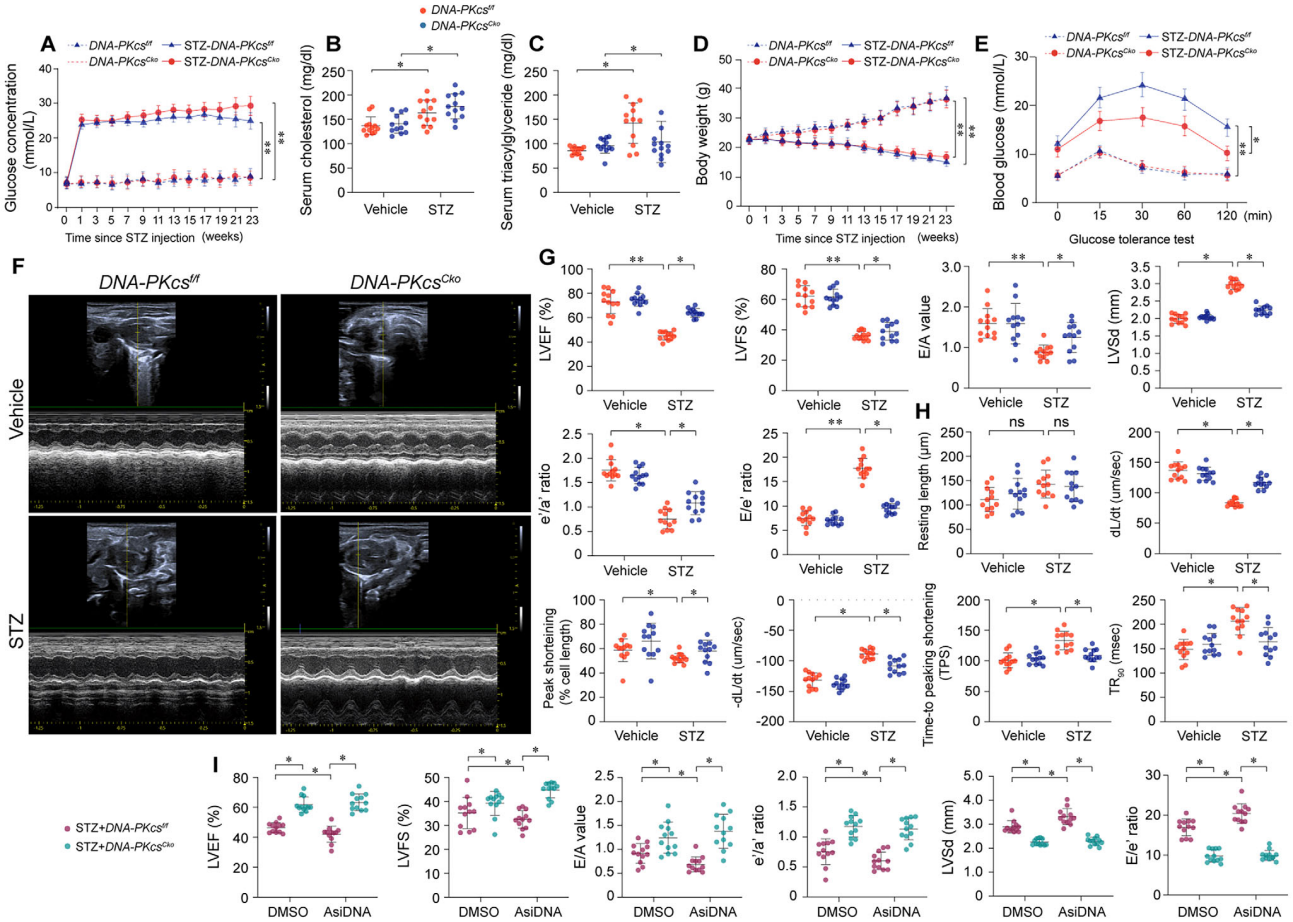
Ablation of DNA‐PKcs improves heart function in the presence of hyperglycemic stress. In vivo, cardiomyocyte‐specific *DNA‐PKcs* knockout (*DNA‐PKcs^Cko^*) and wild‐type *DNA‐PKcs^f/f^* mice were injected intraperitoneally with streptozotocin (STZ, 50 mg kg^−1^ in 0.1 mol L^−1^ citrate buffer) for five consecutive days to induce diabetes. Age‐ and sex‐matched non‐diabetic control mice were injected with an equal volume of PBS. A) Time course of fasting blood glucose levels, measured at baseline (week 0) and periodically over the 24‐week period following STZ or vehicle (citrate buffer) injection. *n* = 8 mice per group and dots in this panel represent the mean values derived from the data of these 8 mice. B,C) Fasting serum cholesterol (B) and triglyceride (C) levels in mice 24 weeks after STZ or vehicle injection. Each group consisted of 4 animals (*n* = 4). For each animal, measurements were repeated three times under the same experimental conditions. In each panel, dots represent individual measurements from animals. Bars represent group means, and error bars indicate ± standard error (SD). D) Time course of body weight changes during the 24‐week study period. *n* = 8 mice per group and dots in this panel represent the mean values derived from the data of these 8 mice. E) Intraperitoneal glucose tolerance test (IPGTT) conducted 9 days after STZ or vehicle treatment in *DNA‐PKcs^Cko^* and *DNA‐PKcs^f/f^* mice. *n* = 8 mice per group and dots in this panel represent the mean values derived from the data of these 8 mice. F,G) Echocardiographic analysis of heart function, including left ventricular ejection fraction (LVEF), fractional shortening (FS), left ventricular systolic dimension (LVSd), left ventricular diastolic dimension (LVDd), early‐to‐late (atrial) mitral flow velocity ratio (E/A), ratio of mitral peak velocity of early filling to early diastolic mitral annular velocity (E/e’), and ratio of diastolic mitral annulus velocities (e’/a’). Each group consisted of 4 animals (*n* = 4). For each animal, measurements were repeated three times under the same experimental conditions. In each panel, dots represent individual measurements from animals. Bars represent group means, and error bars indicate ± standard error (SD). H) Analysis of contractile parameters in acutely isolated single cardiomyocytes from *DNA‐PKcs^Cko^* and *DNA‐PKcs^f/f^* mice, including resting length, peak shortening (PS), maximal velocity of shortening (+dL/dt), time‐to‐peak shortening (TPS), maximal velocity of relengthening (−dL/dt), and time‐to‐90% relengthening (TR90). Each group consisted of independent cell culture experiments (*n* = 4). For each independent cell culture experiment, measurements were repeated three times under the same experimental conditions. In each panel, dots represent individual measurements from independent cell culture experiments. Bars represent group means, and error bars indicate ± standard error (SD). I) Echocardiography measurements of heart function in diabetic *DNA‐PKcs^Cko^* and *DNA‐PKcs^f/f^* mice treated with low‐dose AsiDNA (3 mg kg^−1^) for 24 weeks. Each group consisted of 4 animals (*n* = 4). For each animal or independent cell culture experiment, measurements were repeated three times under the same experimental conditions. In each panel, dots represent individual measurements from animals. Bars represent group means, and error bars indicate ± standard error (SD). *p < 0.05, **p < 0.01, ***p < 0.001, ns, not significant.

Initial metabolic parameters, including fasting plasma glucose, serum cholesterol, triglycerides, body weight, and glucose tolerance, showed no significant differences between *DNA‐PKcs^Cko^* mice and *DNA‐PKcs^f/f^* control mice (**Figure** **3**A–E). Following streptozotocin (STZ) administration, both genotypes exhibited elevated fasting plasma glucose, serum cholesterol, and triglyceride levels, confirming successful diabetes induction (Figure 3A–E and Figure S4C–G, Supporting Information). No significant differences were observed in fasting glucose levels, serum cholesterol, body weight (Figure 3A–E), or serum insulin levels (Figure S4H, Supporting Information) between diabetic *DNA‐PKcs^f/f^* and *DNA‐PKcs^Cko^* mice. This is likely because the deletion of DNA‐PKcs in cardiomyocytes does not influence pancreatic insulin secretion and, consequently, does not directly modulate systemic glucose levels. However, *DNA‐PKcs^Cko^* mice showed a slight reduction in serum triglycerides but not serum cholesterol compared to *DNA‐PKcs^f/f^* controls (Figure 3A–E). Mechanistically, in vitro studies demonstrated that HG‐induced reductions in fatty acid oxidation‐related genes, including *Cpt1a*, *Cpt2*, and *Ppar‐α*, were partially reversed by *DNA‐PKcs* knockdown (Figure S4I, Supporting Information). The improved fatty acid metabolism mediated by *DNA‐PKcs* deletion may contribute to the observed reduction in serum triglycerides—a critical energy source for cardiomyocytes—distinct from cholesterol, which serves as a fundamental component of cell membranes and is predominantly regulated by hepatic metabolism. In contrast, *Ku80* deletion in cardiomyocytes did not significantly affect metabolic parameters (Figure S4C–G, Supporting Information). These findings suggest that *DNA‐PKcs* deficiency minimally impacts systemic metabolic alterations but provides a protective effect on cardiac metabolism under diabetic conditions.

We next investigated the impact of *DNA‐PKcs* deficiency on cardiac function. Echocardiography was employed to assess myocardial contractility and relaxation dynamics in diabetic mice. In *DNA‐PKcs^f/f^* or *Ku80^f/f^* mice, STZ administration impaired cardiac systolic function, as evidenced by a reduced left ventricular ejection fraction (LVEF), decreased fractional shortening (FS), and an enlarged left ventricular systolic dimension (LVSd) (Figure 3F,G and Figure S5A, Supporting Information). Diastolic dysfunction was also observed, as indicated by adverse changes in the early to late transmitral flow velocity ratio (E/A), mitral annulus velocity ratios (e′/a′), and mitral peak velocity to early diastolic mitral annular velocity (E/e′) (Figure 3F,G and Figure S5A, Supporting Information). Remarkably, these functional impairments were reversed in diabetic *DNA‐PKcs^Cko^* mice (Figure 3F,G), while diabetic *Ku80^Cko^* showed no improvement (Figure S5A, Supporting Information).

To further evaluate the effects of *DNA‐PKcs* deficiency on cardiomyocyte contractility, we measured contractile parameters in freshly isolated cardiomyocytes from both non‐diabetic and diabetic mice. STZ treatment did not alter the resting length of cardiomyocytes in either *DNA‐PKcs^f/f^* or *DNA‐PKcs^Cko^* mice (Figure 3H). However, hyperglycemia significantly reduced peak shortening, maximal velocity of shortening (+dL/dt), and time‐to‐peak shortening (TPS) in cardiomyocytes isolated from STZ‐treated *DNA‐PKcs^f/f^* mice (Figure 3H). Additionally, these cardiomyocytes exhibited abnormal relaxation kinetics, including reductions in maximal velocity of relengthening (−dL/dt) and time‐to‐90% relengthening (TR90) (Figure 3H). In contrast, cardiomyocytes from STZ‐treated *DNA‐PKcs^Cko^* mice showed improved contractile and relaxation properties (Figure 3H), which were not observed in *Ku80^Cko^* mice (Figure S5B, Supporting Information).

To further explore the role of DNA‐PKcs in myocardial dysfunction, we treated diabetic *DNA‐PKcs^Cko^* and *DNA‐PKcs^f/f^* mice with AsiDNA, a DNA repair inhibitor that mimics double‐strand breaks (DSBs) and acts as an agonist of DNA‐PKcs. Chronic administration of AsiDNA exacerbated the myocardial contractile/relaxation dysfunction in diabetic *DNA‐PKcs^f/f^* mice compared to DMSO‐treated controls (Figure 3I). However, this detrimental effect was not observed in diabetic *DNA‐PKcs^Cko^* mice, confirming the critical role of DNA‐PKcs in driving hyperglycemia‐induced cardiac dysfunction. In both *Ku80^f/f^* and Ku80*^Cko^* mice, continuous AsiDNA administration further suppressed myocardial function under diabetic conditions (Figure S5C, Supporting Information). Collectively, these results indicate that *DNA‐PKcs* ablation protects against hyperglycemia‐induced cardiac dysfunction, highlighting its potential as a therapeutic target for managing DCM.

### 2.4 *DNA‐PKcs* Deficiency Suppresses Hyperglycemia‐Induced Myocardial Fibrosis and Inflammation

**Figure 4:**
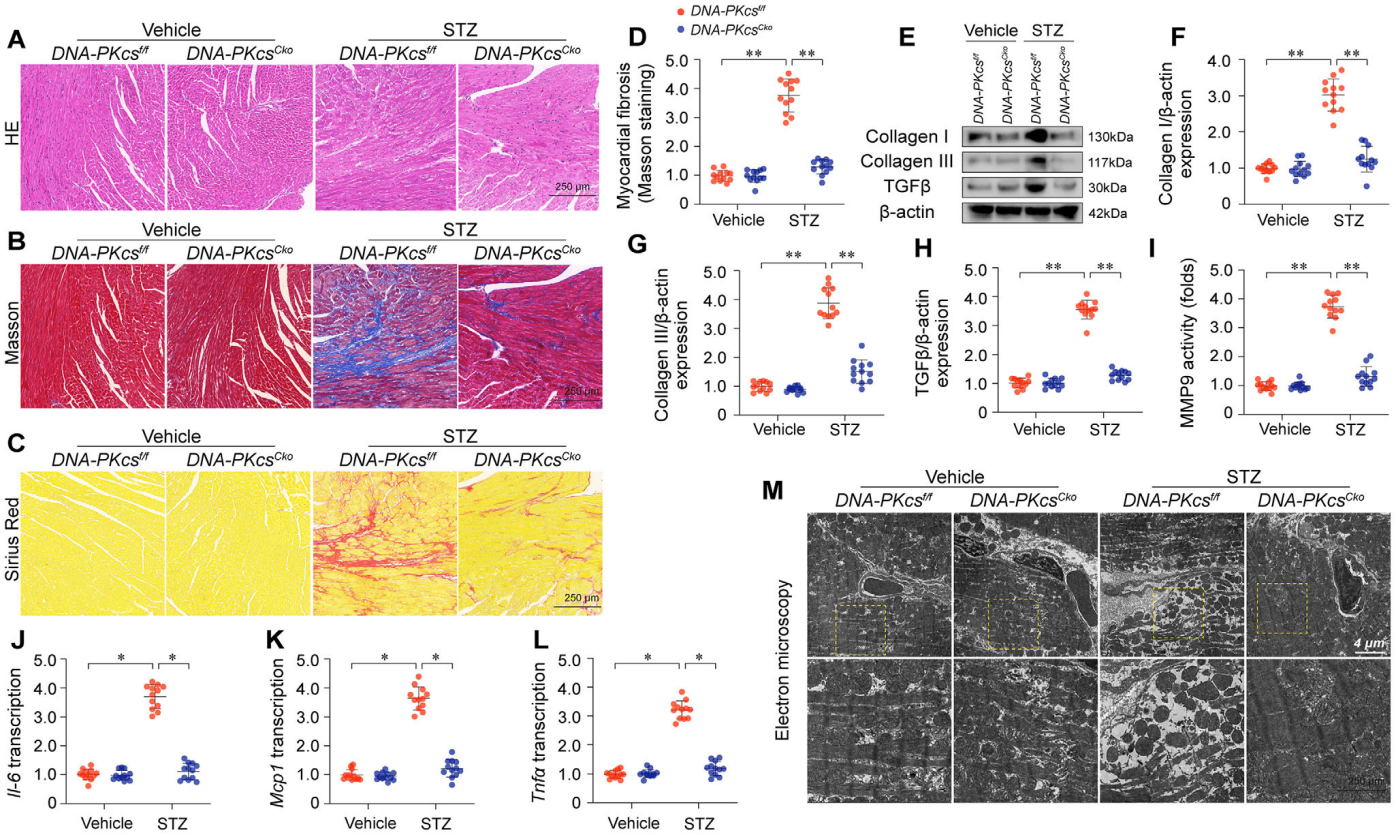
DNA‐PKcs deficiency suppresses hyperglycemia‐induced myocardial structural disorder. In vivo, cardiomyocyte‐specific *DNA‐PKcs* knockout (*DNA‐PKcs^Cko^*) and wild‐type *DNA‐PKcs^f/f^* mice were injected intraperitoneally with streptozotocin (STZ, 50 mg kg^−1^ in 0.1 mol L^−1^ citrate buffer) for five consecutive days to induce diabetes. Age‐ and sex‐matched non‐diabetic control mice were injected with PBS. A) Representative histopathological images (H&E staining) showing myocardial disarray in diabetic mice. B) Representative histopathological images (Masson trichrome staining) illustrating myocardial fibrosis in diabetic mice. C) Representative histopathological images (Sirius Red staining) highlighting myocardial fibrosis in diabetic mice. D) Quantification of Masson trichrome staining showing the extent of myocardial fibrosis. E–H) Western blot analysis of collagen I, collagen III, and TGFβ expression in heart tissue extracts. (I) ELISA analysis of matrix metalloproteinase‐9 (MMP9) activity in heart tissues. J–L) Quantitative PCR (qPCR) analysis of *Il‐6*, *Tnfα*, and *Mcp1* mRNA expression in heart tissues. M) Representative electron microscopy images depicting ultrastructural changes in heart tissue from diabetic mice. Each group consisted of 4 animals or 4 independent cell culture experiments (*n* = 4). For each animal or independent cell culture experiment, measurements were repeated three times under the same experimental conditions. In each panel, dots represent individual measurements from animals or independent cell culture experiments. Bars represent group means, and error bars indicate ± standard error (SD). *p < 0.05, **p < 0.01, ***p < 0.001, ns, not significant.

In order to better understand the cardioprotective capabilities conferred by *DNA‐PKcs* deficiency in the context of DCM, we conducted an analysis of cardiac structural integrity using H&E, Masson trichrome, and Sirius Red staining. STZ was observed to induce myocardial disarray (**Figures** **4**A and S6A, Supporting Information) and fibrosis (Figure 4B–D and Figure S6B–D, Supporting Information) in both *DNA‐PKcs^f/f^* and *Ku80^f/f^* mice, effects that were notably mitigated in *DNA‐PKcs^Cko^* mice but remained unaltered in *Ku80^Cko^* mice. Protein analysis using western blotting revealed an upregulated expression of collagen I/III and transforming growth factor‐β (TGFβ) in both *DNA‐PKcs^f/f^* and *Ku80^f/f^* mice (Figure 4E–H and Figure S6E–G, Supporting Information). Interestingly, this hyperglycemia‐mediated upregulation was prevented by the deletion of *DNA‐PKcs*, but not *Ku80* (Figure 4E–H and Figure S6E–G, Supporting Information). This was further corroborated by an ELISA assay, which demonstrated that matrix metallopeptidase 9 (MMP9) activity was significantly elevated in response to STZ injection in *DNA‐PKcs^f/f^* and *Ku80^f/f^* mice (Figure 4I and Figure S6H, Supporting Information), an alteration that was undetectable in *DNA‐PKcs^Cko^* mice but still present in *Ku80^Cko^* mice. qPCR analysis revealed an upregulation of inflammatory markers, such as *Il‐6*, *Tnfα*, and *Mcp1*, in STZ‐treated *DNA‐PKcs^f/f^* and *Ku80^f/f^* mice (Figure 4J–L and Figure S6J–L, Supporting Information). This upsurge was effectively prevented by the ablation of *DNA‐PKcs*, but not *Ku80* (Figure 4J–L and Figure S6I, Supporting Information). To identify the source of the inflammatory factors, RNA was isolated from high‐glucose (HG)‐treated cardiomyocytes. qPCR analysis revealed that the HG‐induced upregulation of inflammatory factors was attenuated by *DNA‐PKcs* deletion (Figure S6J, Supporting Information).

Subsequent ultrastructural analysis of the myocardium, conducted using electron microscopy, revealed that STZ treatment was associated with disordered myofibrils, mitochondrial swelling, and a reduced number of mitochondria in heart samples from *DNA‐PKcs^f/f^* mice (Figure 4M). Remarkably, these myocardial ultrastructural changes induced by STZ were largely prevented in cardiomyocytes that deletion of *DNA‐PKcs* (Figure 4M). These results strongly suggest that *DNA‐PKcs* deficiency may serve as an effective protective mechanism against DCM‐caused myocardial structural disorder.

### 2.5 *DNA‐PKcs* Deletion Suppresses Ferroptosis in Diabetic Cardiomyopathy

**Figure 5:**
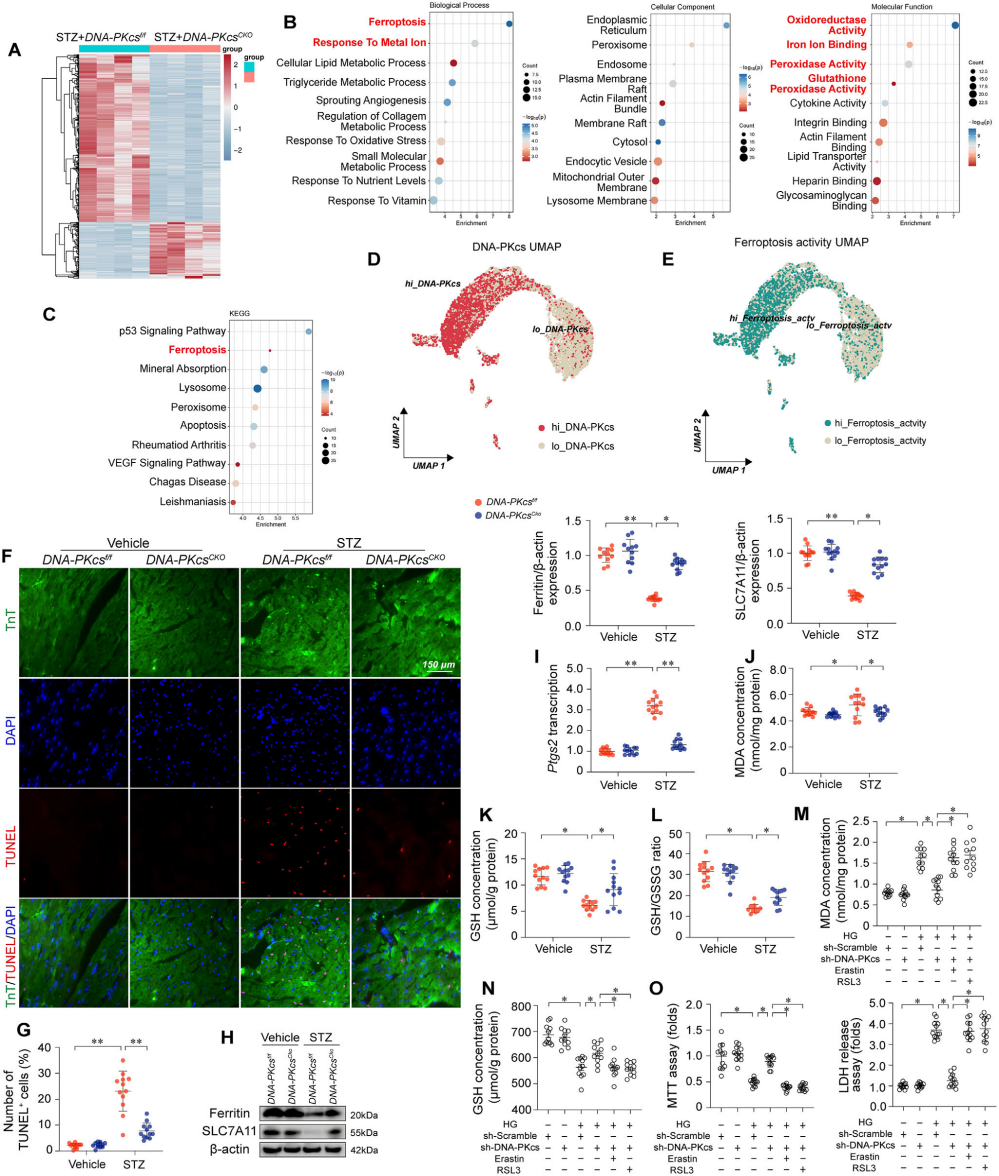
DNA‐PKcs deletion suppresses ferroptosis in diabetic cardiomyopathy. In vivo, cardiomyocyte‐specific *DNA‐PKcs* knockout (*DNA‐PKcs^Cko^*) and wild‐type *DNA‐PKcs^f/f^* mice were injected intraperitoneally with streptozotocin (STZ, 50 mg kg^−1^ in 0.1 mol L^−1^ citrate buffer) for five consecutive days to induce diabetes. Age‐ and sex‐matched non‐diabetic control mice were injected with PBS. In vitro, HL‐1 cells were transduced with shRNA targeting DNA‐PKcs (sh/DNA‐PKcs) or with scramble control (sh/scramble), and then cultured in high‐glucose (HG, 30 mmol L^−1^) medium for 48 h to mimic hyperglycemic stress. Cells incubated in normal glucose (NG, 5.5 mmol L^−1^) medium served as controls. A) RNA‐seq analysis of differentially expressed genes in STZ‐treated *DNA‐PKcs^Cko^* and STZ‐injected *DNA‐PKcs^f/f^* mice. B,C) KEGG and GO analyses of differentially expressed genes. D,E) Single‐cell RNA sequencing (scRNA‐seq) data from the Genome Sequence Archive in the BIG Data Center (http://bigd.big.ac.cn/, accession code CRA007245) was used to assess the correlation between DNA‐PKcs expression levels and ferroptosis pathway activity scores. F,G) Representative images of TUNEL staining in heart tissues from vehicle‐ and STZ‐treated mice, and corresponding quantification data. DAPI was used for nuclear staining. H) Western blot analysis of ferritin and SLC7A11 protein expression in heart tissues. I) qPCR analysis of *Ptgs2* transcription in heart tissues. J–L) ELISA‐based quantification of malondialdehyde (MDA), glutathione (GSH), and the GSH/GSSG ratio in heart tissues. M,N) ELISA quantification of MDA levels, GSH levels, and the GSH/GSSG ratio in HL‐1 cells. HL‐1 cells were treated with ferrostatin‐1 (Fer‐1) or deferoxamine (DFO) prior to HG exposure. O) Cell death was assessed by MTT assay and lactate dehydrogenase (LDH) release in HL‐1 cells treated with Fer‐1 or DFO before HG exposure. Each group consisted of 4 animals or 4 independent cell culture experiments (*n* = 4). For each animal or independent cell culture experiment, measurements were repeated three times under the same experimental conditions. In each panel, dots represent individual measurements from animals or independent cell culture experiments. Bars represent group means, and error bars indicate ± standard error (SD). *p < 0.05 **p < 0.01, ***p < 0.001, ns, not significant.

To elucidate the molecular mechanisms underlying the protective effect of *DNA‐PKcs* deletion in DCM, we employed an unbiased RNA‐seq approach. Our analysis revealed that *DNA‐PKcs* deficiency significantly altered the transcriptional landscape of 1297 genes in the heart following STZ intervention (log_2_FC>1.0, p<0.05). The differential expression analysis depicted in the volcano plot identified 968 upregulated genes and 329 downregulated genes in *DNA‐PKcs*‐deficient diabetic hearts (**Figure** **5**A). GO Biological Process analysis indicated that these differentially expressed genes were predominantly involved in pathways related to ferroptosis and metal ion homeostasis (Figure 5B). GO Molecular Function analysis further highlighted that *DNA‐PKcs* deficiency mainly affected oxioreductase activity, iron ion binding, peroxidase activity, and glutathione peroxidase activity—key molecular functions implicated in oxidative stress and ferroptosis regulation (Figure 5B). Consistent with these findings, KEGG pathway analysis revealed that genes orchestrating ferroptosis were principally impacted by *DNA‐PKcs* deletion in the diabetic heart (Figure 5C), suggesting a pivotal role for DNA‐PKcs in modulating ferroptotic pathways under hyperglycemic conditions. To further investigate the relationship between DNA‐PKcs and ferroptosis, we performed single‐cell RNA sequencing (scRNA‐seq) analyses using publicly available data from the Genome Sequence Archive in the BIG Data Center (http://bigd.big.ac.cn/, accession code CRA007245). This dataset comprises nuclear fractions of cardiac cells from six healthy controls (16 490 cells) and six high‐fat diet (HFD)/STZ‐induced diabetic mice (16 095 cells). Our results demonstrated a strong correlation between DNA‐PKcs expression levels (Figure 5D) and ferroptosis pathway activity scores (Figure 5E) across different cell types, suggesting that DNA‐PKcs expression closely tracks with ferroptotic activity in the diabetic heart.

Given the growing recognition of ferroptosis as a key contributor to DCM, we investigated whether *DNA‐PKcs* deletion could modulate ferroptosis in the diabetic heart. TUNEL staining revealed that hyperglycemic stress induced significant cardiomyocyte death, an effect that was notably reversed in *DNA‐PKcs*‐deficient mice (Figure 5F,G). Western blot analysis further demonstrated a substantial downregulation of ferroptosis‐related proteins, such as solute carrier family 7 member 11 (SLC7A11) and ferritin, in the hearts of diabetic *DNA‐PKcs^f/f^* mice compared to non‐diabetic controls (Figure 5H). This downregulation was partially alleviated in *DNA‐PKcs^Cko^* mice (Figure 5H). Additionally, *Ptgs2*, a marker of ferroptosis, was transcriptionally upregulated in diabetic *DNA‐PKcs^f/f^* hearts and returned to near‐normal levels in *DNA‐PKcs*‐deficient mice (Figure 5I), indicating that *DNA‐PKcs* deficiency offers protection against ferroptosis in the context of diabetic heart injury.

Lipid peroxidation, a hallmark of ferroptosis, was assessed by measuring malondialdehyde (MDA) levels and the glutathione (GSH)/oxidized glutathione (GSSG) ratio. Diabetic *DNA‐PKcs^f/f^* mice exhibited significantly elevated MDA content and a reduced GSH/GSSG ratio, indicative of increased oxidative stress and lipid peroxidation (Figure 5J–L). In contrast, *DNA‐PKcs^Cko^* mice showed a marked reduction in MDA levels and an improved GSH/GSSG ratio, suggesting a suppression of lipid peroxidation and oxidative stress (Figure 5J–L). To further explore the role of DNA‐PKcs in regulating ferroptosis, we employed an in vitro model using HL‐1 cardiomyocytes transfected with short hairpin RNA (shRNA) targeting DNA‐PKcs (sh/DNA‐PKcs) and exposed the cells to HG. *DNA‐PKcs* knockdown significantly decreased lipid peroxidation markers (Figure 5M,N) and improved cell viability (Figure 5O) compared to control shRNA‐treated cells. However, co‐treatment with either the ferroptosis inducer erastin or RSL3 reversed the protective effects of *DNA‐PKcs* knockdown, confirming that DNA‐PKcs mediates ferroptosis under hyperglycemic conditions (Figure 5M–O). These findings demonstrate that *DNA‐PKcs* deletion suppresses ferroptosis in diabetic cardiomyopathy, thereby preserving cardiomyocyte viability and mitochondrial integrity under hyperglycemic stress.

### 2.6 Re‐Activation of Ferroptosis Abolishes the Cardioprotective Effects of *DNA‐PKcs* Deletion In Vivo and In Vitro

**Figure 6:**
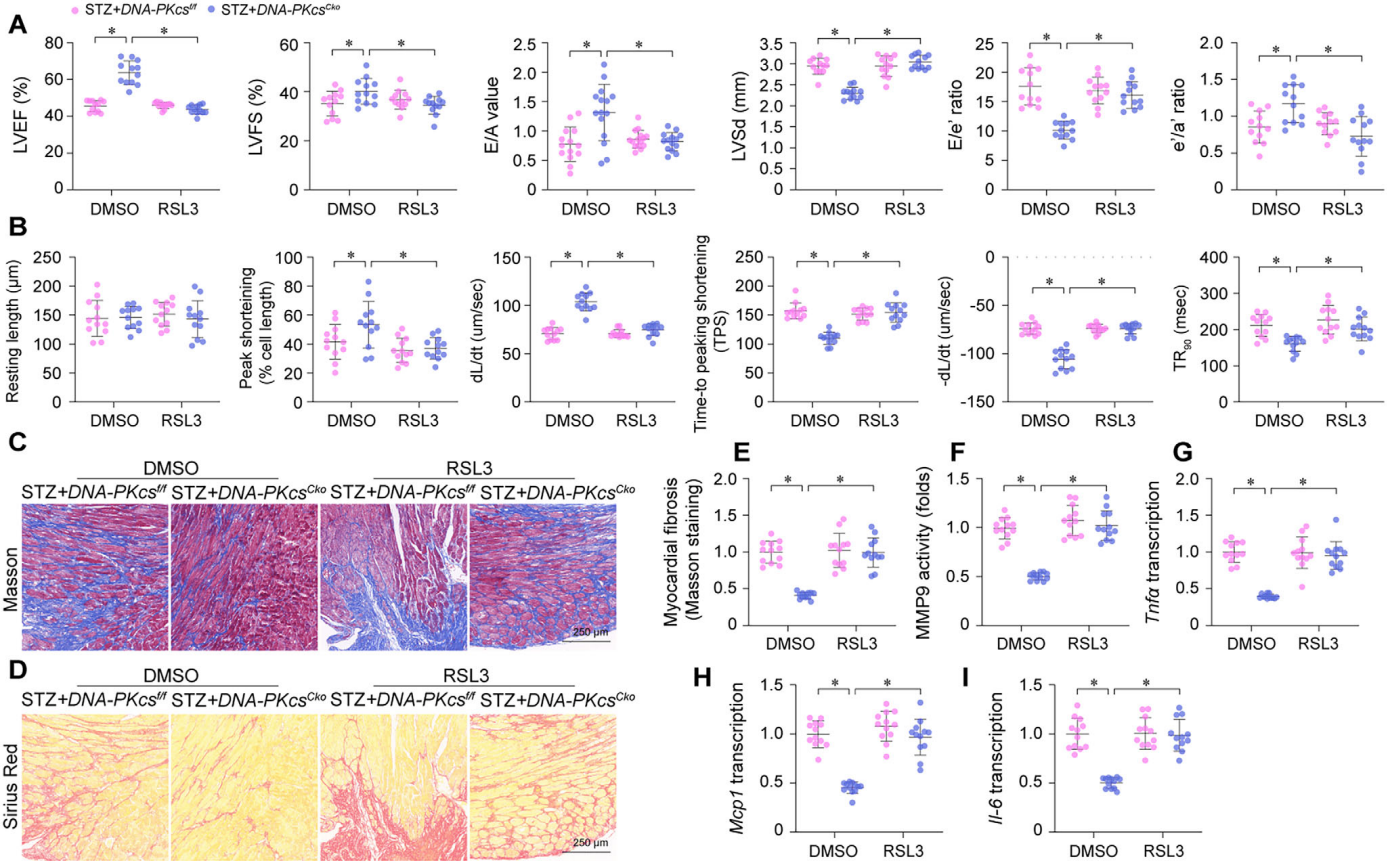
Re‐activation of ferroptosis abolishes the cardioprotective effects of DNA‐PKcs deletion in vivo and in vitro. In vivo, cardiomyocyte‐specific *DNA‐PKcs* knockout (*DNA‐PKcs^Cko^*) and wild‐type *DNA‐PKcs^f/f^* mice were injected intraperitoneally with streptozotocin (STZ, 50 mg kg^−1^ in 0.1 mol L^−1^ citrate buffer) for five consecutive days to induce diabetes. Age‐ and sex‐matched non‐diabetic control mice were injected with PBS. Diabetic *DNA‐PKcs^Cko^* and *DNA‐PKcs^f/f^* mice were subsequently injected intraperitoneally with RSL3 (10 mg kg^−1^) for 24 weeks to induce ferroptosis. A) Echocardiographic analysis of heart function, including left ventricular ejection fraction (LVEF), fractional shortening (FS), left ventricular systolic dimension (LVSd), left ventricular diastolic dimension (LVDd), early‐to‐late (atrial) mitral flow velocity ratio (E/A), ratio of mitral peak velocity of early filling to early diastolic mitral annular velocity (E/e’), and ratio of diastolic mitral annulus velocities (e’/a’). B) Analysis of contractile parameters in acutely isolated single cardiomyocytes from *DNA‐PKcs^Cko^* and *DNA‐PKcs^f/f^* mice, including peak shortening (PS), maximal velocity of shortening (+dL/dt), time‐to‐peak shortening (TPS), maximal velocity of relengthening (−dL/dt), and time‐to‐90% relengthening (TR90). C) Representative histopathological images (Masson trichrome staining) showing myocardial fibrosis in diabetic mice. D) Representative histopathological images (Sirius Red staining) illustrating myocardial fibrosis in diabetic mice. E) Quantification of myocardial fibrosis from Masson trichrome staining. F) ELISA‐based analysis of matrix metalloproteinase‐9 (MMP9) activity in heart tissues. G–I) Quantitative PCR (qPCR) analysis of *Il‐6*, *Tnfα*, and *Mcp1* mRNA expression in heart tissues. Each group consisted of 4 animals or 4 independent cell culture experiments (*n* = 4). For each animal or independent cell culture experiment, measurements were repeated three times under the same experimental conditions. In each panel, dots represent individual measurements from animals or independent cell culture experiments. Bars represent group means, and error bars indicate ± standard error (SD). *p < 0.05, **p < 0.01, ***p < 0.001, ns, not significant.

To elucidate the role of DNA‐PKcs in modulating cardiac function via ferroptosis, we administered RSL3, a potent inducer of ferroptosis, to diabetic *DNA‐PKcs^Cko^* mice. Subsequently, we evaluated cardiac function using echocardiography. Despite the enhancement of cardiac contractility and relaxation following *DNA‐PKcs* deletion under STZ treatment, RSL3 administration nullified this beneficial effect (**Figure** **6**A). Similarly, contractile parameters of freshly isolated cardiomyocytes from STZ‐treated *DNA‐PKcs^Cko^* mice were preserved compared to those from STZ‐treated *DNA‐PKcs^f/f^* mice (Figure 6B). However, RSL3 treatment negated the protective effect of *DNA‐PKcs* deletion on cardiomyocyte contractility in the context of STZ treatment (Figure 6B). Furthermore, we observed STZ‐induced myocardial fibrosis in *DNA‐PKcs^f/f^* mice, but not in *DNA‐PKcs^Cko^* mice (Figure 6C–E). Notably, reactivation of ferroptosis via RSL3 treatment counteracted the anti‐fibrotic effect of *DNA‐PKcs* ablation in the diabetic heart, as demonstrated by Masson's trichrome and Sirius Red staining (Figure 6C–E). These observations were corroborated by ELISA analysis of MMP9 activity (Figure 6F). Interestingly, while *DNA‐PKcs^Cko^* hearts exhibited a diminished inflammatory response, as indicated by quantitative PCR analysis of pro‐inflammatory factors compared to *DNA‐PKcs^f/f^* hearts, this anti‐inflammatory effect was annulled by RSL3 (Figure 6G–I). Collectively, these findings corroborate that ferroptosis operates downstream of DNA‐PKcs and that the induction of ferroptosis negates the cardioprotective effects of *DNA‐PKcs* deletion in the context of diabetic cardiomyopathy.

### 2.7 Co‐IP‑Based Proteomics Analysis of DNA‐PKcs

**Figure 7:**
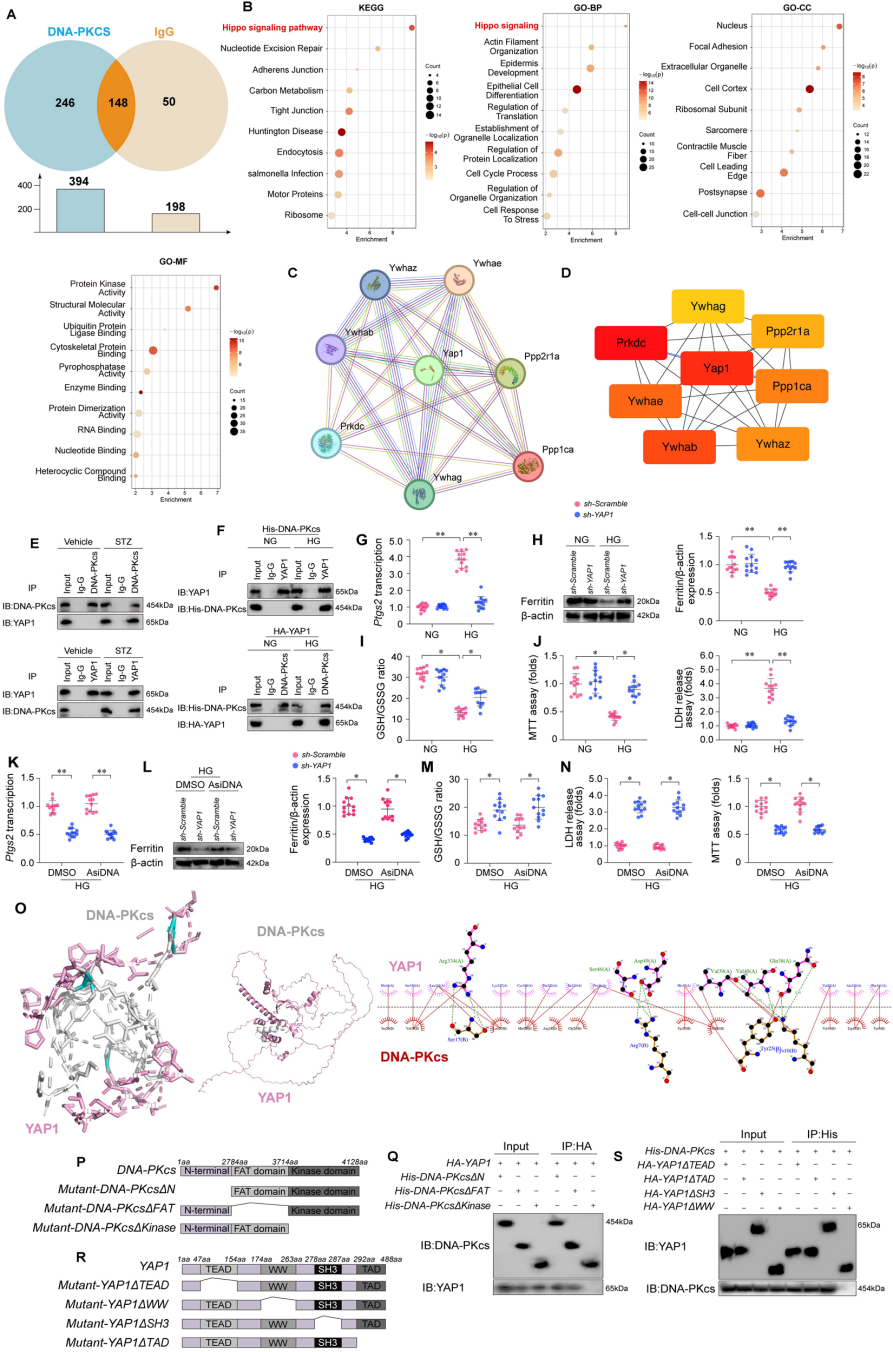
Co‐immunoprecipitation (Co‐IP)‐based proteomics analysis of DNA‐PKcs interactions. In vivo, cardiomyocyte‐specific *DNA‐PKcs* knockout (*DNA‐PKcs^Cko^*) and wild‐type *DNA‐PKcs^f/f^* mice were injected intraperitoneally with streptozotocin (STZ, 50 mg kg^−1^ in 0.1 mol L^−1^ citrate buffer) for five consecutive days to induce diabetes. Age‐ and sex‐matched non‐diabetic control mice were injected with PBS. Diabetic *DNA‐PKcs^Cko^* and *DNA‐PKcs^f/f^* mice were subsequently injected intraperitoneally with RSL3 (10 mg kg^−1^) for 24 weeks to induce ferroptosis. A) A Co‐IP‐based proteomics assay was performed to identify DNA‐PKcs‐interacting proteins. The Venn diagram illustrates the overlap of proteins between the IgG control and DNA‐PKcs groups. B) KEGG and GO analyses of DNA‐PKcs‐interacting proteins. C) Protein‐protein interaction (PPI) network analysis of DNA‐PKcs‐specific binding proteins, focusing on a curated gene list associated with the Hippo pathway. D) CytoHubba plugin was used to score the PPI network. Higher scores, indicated by red color, represent proteins that are more central within the network. E) Immunoprecipitates of DNA‐PKcs or YAP1 from heart tissues of mice with hyperglycemia‐induced diabetic cardiomyopathy were immunoblotted as indicated. F) His‐tagged DNA‐PKcs or HA‐tagged YAP1 was transfected into HL‐1 cells, followed by a Co‐IP assay to assess protein interactions. G) HL‐1 cells were treated with shRNA targeting YAP1 (sh/YAP1) before high glucose (HG) exposure, and qPCR analysis was performed to assess Ptgs2 transcription. H) HL‐1 cells were treated with sh/YAP1 before HG exposure, and Western blotting was used to determine ferritin expression in heart tissues. I) ELISA kits were used to analyze GSH/GSSG ratios in HL‐1 cells treated with sh/YAP1 prior to HG exposure. J) Cell death was measured in HL‐1 cells treated with sh/YAP1 before HG exposure using MTT and LDH release assays. K) HL‐1 cells were treated with sh/YAP1 before AsiDNA treatment, and qPCR analysis was used to assess Ptgs2 transcription. L) Western blot analysis of ferritin expression in heart tissues from HL‐1 cells treated with sh/YAP1 prior to AsiDNA treatment. M) ELISA kits were used to determine GSH/GSSG ratios in HL‐1 cells treated with sh/YAP1 before AsiDNA treatment. N) Cell death was measured using MTT and LDH release assays in HL‐1 cells treated with sh/YAP1 before AsiDNA treatment. O) Molecular docking analysis of DNA‐PKcs and YAP1 showing potential binding sites, highlighted in different colors. P) Mapping of functional regions in DNA‐PKcs involved in binding to YAP1. Q) Immunoprecipitation and immunoblotting were applied to evaluate interactions between specific DNA‐PKcs mutants and YAP1. R) Mapping of functional regions in YAP1 involved in interaction with DNA‐PKcs. S) Immunoprecipitation and immunoblotting were used to assess interactions between region‐specific DNA‐PKcs and YAP1 mutants in HL‐1 cells. Each group consisted of 4 animals or 4 independent cell culture experiments (*n* = 4). For each animal or independent cell culture experiment, measurements were repeated three times under the same experimental conditions. In each panel, dots represent individual measurements from animals or independent cell culture experiments. Bars represent group means, and error bars indicate ± standard error (SD). *p < 0.05, **p < 0.01, ***p < 0.001, ns, not significant.

While the functional role of DNA‐PKcs in hyperglycemia‐induced ferroptosis and myocardial dysfunction is well‐established, the molecular pathways through which DNA‐PKcs regulates ferroptosis remain to be elucidated. To uncover the molecular mechanisms underlying the activation of ferroptosis following DNA‐PKcs upregulation, we employed a co‐immunoprecipitation (Co‐IP)‐based proteomics assay to identify DNA‐PKcs‐interacting proteins. Immunoglobulin G (IgG) was used as a negative control for Co‐IP. Our analysis identified 394 proteins within the DNA‐PKcs‐IP group, while 198 proteins were detected in the IgG control group (**Figure** **7**A). Notably, 148 proteins were present in both groups. KEGG and GO enrichment analyses revealed that the proteins specifically interacting with DNA‐PKcs were predominantly enriched in the Hippo signaling pathway (Figure 7B). We further intersected the DNA‐PKcs‐specific binding proteins with a curated gene list associated with the Hippo pathway, identifying seven relevant proteins (Figure 7C). Protein‐protein interaction (PPI) network analysis of these seven proteins revealed that YAP1 occupied a central position within the network (Figure 7C). To further validate YAP1's role, we applied the CytoHubba plugin to score the PPI network (with higher scores indicated by red color, representing more central proteins in the network) (Figure 7D). YAP1 scored the highest, confirming its key position in the DNA‐PKcs‐mediated regulation of ferroptosis (Figure 7D).

Co‐immunoprecipitation (Co‐IP) assays confirmed that DNA‐PKcs interacts with YAP1 (Figure 7E), but not with MST1 (Figure S7A, Supporting Information) or LATS2 (Figure S7B, Supporting Information), the classical components of the Hippo pathway, under hyperglycemic conditions. Furthermore, exogenous His‐tagged DNA‐PKcs was successfully pulled down by YAP1 (Figure 7F), but not by MST1 (Figure S7C, Supporting Information) or LATS2 (Figure S7D, Supporting Information), in a hyperglycemic environment. Functional assays further demonstrated that knockdown of *YAP1* via shRNA (sh‐YAP1) (Figure 7G–J), in contrast to MST1 (sh‐MST1) (Figure S7E–I, Supporting Information) or LATS2 (sh‐LATS2) knockdown (Figure S7J–N, Supporting Information), significantly alleviated hyperglycemia‐induced cardiomyocyte damage and the induction of ferroptosis. Similarly, in HL‐1 cells transfected with sh‐YAP1, AsiDNA failed to induce ferroptosis (Figure 7K–M) or cardiomyocyte damage (Figure 7N). These findings highlight the indispensable role of YAP1 in DNA‐PKcs‐mediated ferroptosis, positioning YAP1 as a key regulator in the context of DCM.

Molecular docking assays have delineated the requisite regions instrumental for the DNA‐PKcs‐YAP1 interaction, characterized by a calculated minimum binding energy of −25.1 kcal·mol^−1^ (Figure 7O). This finding underscores the specificity inherent in this interaction. To elucidate the molecular underpinnings of the DNA‐PKcs and YAP1 interaction, we scrutinized the domains imperative for their cross‐linking. Transfection of HL‐1 cells with an assortment of *DNA‐PKcs* deletion mutants divulged that the Kinase domain of DNA‐PKcs is indispensable for YAP1 binding (Figure 7P,Q). On the other hand, HA‐tagged YAP1 devoid of the Tryptophan‐Tryptophan (WW) domain (HA‐YAP1ΔWW) manifested a disruption in its DNA‐PKcs binding capability (Figure 7R,S). This evidence infers that the Kinase domain of DNA‐PKcs and the WW domain of YAP1 are integral to their interactive capacity. Notably, the transfection of either YAP1ΔWW (Figure S8A–D, Supporting Information) or His‐DNA‐PkcsΔKinase (Figure S8E–H, Supporting Information) was able to alleviate hyperglycemia‐induced ferroptosis and cardiomyocyte damage, underscoring the pivotal role that the DNA‐PKcs/YAP1 interaction plays in the modulation of hyperglycemia‐associated ferroptosis.

### 2.8 DNA‐PKcs Phosphorylates YAP1 at Thr226 and Cause its Nuclear Localization

**Figure 8:**
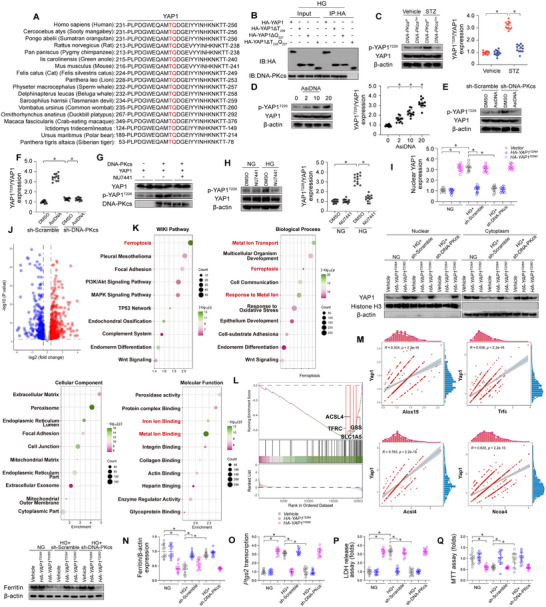
DNA‐PKcs phosphorylates YAP1 at Thr226 and promotes its nuclear localization. A) Amino acid sequence alignment of YAP1 across various species. B) HL‐1 cells were transfected with different HA‐tagged YAP1 constructs, including full‐length YAP1 (HA‐YAP1), YAP1 lacking Thr226 (HA‐YAP1ΔT226), Gln227 (HA‐YAP1ΔQ227), or both Thr226 and Gln227 (HA‐YAP1ΔT226Q227). After high glucose exposure, HA immunoprecipitates were collected and immunoblotted to assess the interaction between DNA‐PKcs and HA‐YAP1. C) YAP1 phosphorylation was assessed in heart tissues from mice subjected to STZ‐induced diabetic cardiomyopathy. D) YAP1 phosphorylation was measured in HL‐1 cells treated with AsiDNA. E,F) HL‐1 cells were treated with shRNA targeting YAP1 (sh/YAP1) prior to AsiDNA treatment, and YAP1 phosphorylation was assessed. G) In vitro kinase assay: recombinant mouse DNA‐PKcs and recombinant mouse YAP1 were incubated together in kinase assay buffer with ATP in the presence or absence of NU7441. YAP1 phosphorylation and DNA‐PKcs levels were assessed by Western blotting. H) HL‐1 cells were treated with NU7441 under high glucose conditions, and YAP1 phosphorylation levels were determined by Western blotting. I) HL‐1 cells were transfected with HA‐tagged YAP1 constructs, including phosphorylation‐defective HA‐YAP1^T226A^ and phosphorylation‐mimetic HA‐YAP1^T226D^. After high glucose exposure, YAP1 protein expression in the cytoplasm and nucleus was analyzed by Western blotting. Histone H3 and β‐actin were used as loading controls. J) Volcano plot of RNA‐seq data from the GSE110268 database, which includes isogenic YAP knockout human embryonic stem cells (hESCs) generated via CRISPR/Cas9‐mediated gene editing. K) WIKI pathway and Gene Ontology (GO) analyses of altered genes from the GSE110268 database. L) Gene Set Enrichment Analysis (GSEA) plots of altered genes from the GSE110268 database. M) Single‐cell RNA sequencing (scRNA‐seq) data from the Genome Sequence Archive (http://bigd.big.ac.cn/, accession code CRA007245) was analyzed to assess the correlation between YAP1 expression levels and ferroptosis‐related gene expression. N) HL‐1 cells were transfected with phosphorylation‐defective HA‐YAP1^T226A^ and phosphorylation‐mimetic HA‐YAP1^T226D^ constructs. After high glucose exposure, ferritin expression was determined by Western blotting. O) HL‐1 cells transfected with YAP1 constructs were exposed to high glucose, and GSH/GSSG ratios were analyzed using ELISA kits. P,Q) HL‐1 cells transfected with YAP1 constructs were exposed to high glucose, and cell death was measured by MTT assay and lactate dehydrogenase (LDH) release assay. Each group consisted of 4 animals or 4 independent cell culture experiments (*n* = 4). For each animal or independent cell culture experiment, measurements were repeated three times under the same experimental conditions. In each panel, dots represent individual measurements from animals or independent cell culture experiments. Bars represent group means, and error bars indicate ± standard error (SD). *p < 0.05, **p < 0.01, ***p < 0.001, ns, not significant.

The DNA‐PKcs enzyme has been reported to interact with proteins, specifically identifying and binding to serine or threonine residues that are followed by a glutamine (SQ/TQ) motif. This interaction triggers the phosphorylation of serine and threonine residues within the SQ/TQ motifs. In our research, we have identified an evolutionarily conserved SQ/TQ motif within the WW domain of the YAP1 protein (**Figure** **8**A). Intriguingly, an HA‐tagged variant of YAP1 that lacks the TQ motif demonstrated an inhibited ability to bind to DNA‐PKcs (Figure 8B). This finding suggests that the TQ motif of YAP1 may be recognized and targeted by DNA‐PKcs.

Since the kinase domain of DNA‐PKcs was required for its interaction with the TQ motif of YAP1, we pursued the question of whether the interaction between DNA‐PKcs and YAP1 could induce YAP1 phosphorylation. To facilitate this investigation, we generated a phospho‐specific antibody for YAP1 at the Thr226 site. Under non‐stressed conditions, we observed little phosphorylation of endogenous YAP1 either in vivo (Figure 8C) or in vitro (Figure S9A, Supporting Information). However, in response to hyperglycemic stress, we detected YAP1 phosphorylation at Thr226 both in vivo (Figure 8C) and in vitro (Figure S9A, Supporting Information). This phosphorylation was inhibited by the deletion of *DNA‐PKcs* (Figure 8C and Figure S9A, Supporting Information). Under similar conditions, treatment with AsiDNA triggered YAP1 phosphorylation in a dose‐dependent manner, primarily at the Thr226 site (Figure 8D), and this effect was abolished by deletion of *DNA‐PKcs* (Figure 8E,F). Our in vitro kinase assay further revealed that DNA‐PKcs enhances YAP1 phosphorylation at Thr226 (Figure 8G). Computational analysis suggested that DNA‐PKcs could facilitate YAP1 phosphorylation at the Thr226 site.^[^ ^24^ ^]^ To establish whether the kinase activity of DNA‐PKcs is essential for YAP1 phosphorylation, we utilized the DNA‐PKcs kinase activity inhibitor NU7441. Notably, in hyperglycemia‐exposed HL‐1 cells, NU7441 effectively abolished DNA‐PKcs–induced YAP1 phosphorylation at Thr226 (Figure 8H).

Prior research has indicated a correlation between YAP1 phosphorylation at Ser127 and its cytoplasmic retention and degradation, while YAP1 phosphorylation at Tyr357 prompts its nuclear localization.^[^ ^25^ ^]^ Given the stark contrast in outcomes depending on the phosphorylation sites of YAP1, we delved deeper into the impact of YAP1 phosphorylation at Thr226 on its subcellular location. We accomplished this by constructing two mutants: the phosphorylation‐defective HA‐YAP1^T226A^, and the phosphorylation‐mimetic HA‐YAP1^T226D^. We observed that hyperglycemia induced the translocation of YAP1 from the cytoplasm to the nucleus, an effect that could be inhibited by the deletion of *DNA‐PKcs* or HA‐YAP1^T226A^ transfection (Figure 8I and Figure S9B, Supporting Information). Intriguingly, in HL‐1 cells transfected with the HA‐YAP1^T226D^ mutant, the deletion of sh/DNA‐PKcs did not prevent the hyperglycemia‐induced nuclear retention of YAP1 (Figure 8I and Figure S9B, Supporting Information). This evidence substantiates that YAP1 phosphorylation at Thr226 enhances its nuclear expression.

In the nucleus, YAP1 primarily functions as a transcriptional co‐activator, interacting predominantly with the TEAD family of transcription factors to regulate gene expression, particularly ferroptosis‐related genes,^[^ ^26^ ^]^ although this molecular mechanism has not been confirmed in DCM setting. In our study, we selected potential YAP‐TEAD gene targets from the GSE110268 dataset, which contains RNA sequencing data from isogenic *YAP* knockout human embryonic stem cells (hESCs) generated via CRISPR/Cas9 gene editing (Figure 8J). WIKI pathway analysis revealed that the differentially expressed genes were associated with ferroptosis (Figure 8K). GO Biological Process analysis further demonstrated that *YAP* deletion impacted metal ion transport, ferroptosis, and response to metal ions. GO Molecular Function analysis indicated that iron ion binding and metal ion binding were controlled by YAP. Moreover, GSEA showed that *YAP* deficiency was associated with a decline in the expression of key ferroptosis regulators, including *Acsl4*, *Gss*, *Tfrc*, and *Slc1A5* (Figure 8L). To further investigate the relationship between YAP1 and ferroptosis, we re‐analyzed scRNA‐seq data from the Genome Sequence Archive in the BIG Data Center (http://bigd.big.ac.cn/, accession code CRA007245), which includes nuclear fractions of cardiac cells from six healthy controls (16 490 cells) and six high‐fat diet (HFD)/STZ‐induced diabetic mice (16 095 cells). The results revealed a strong correlation between YAP1 expression levels and ferroptosis‐related genes, such as *Alox15*, *Acsl4*, *Tfrc*, and *Ncoa4* (Figure 8M).

Validation experiments confirmed that HG‐induced ferroptosis (Figure 8N,O) and cardiomyocyte damage (Figure 8P,Q) were significantly attenuated by sh‐DNA‐PKcs or HA‐YAP1^T226A^ transfection. Conversely, in HL‐1 cells transfected with HA‐YAP1^T226D^, knockdown of *DNA‐PKcs* failed to inhibit HG‐induced ferroptosis (Figure 8N,O) and cardiomyocyte damage (Figure 8P,Q). These findings demonstrate that DNA‐PKcs recognizes the TQ motif of YAP1 and phosphorylates it at Thr226, resulting in the nuclear retention of YAP1, which in turn drives ferroptosis.

### 2.9 Repression of YAP Thr226 Phosphorylation Attenuates Hyperglycemia‐Mediated Heart Dysfunction

**Figure 9:**
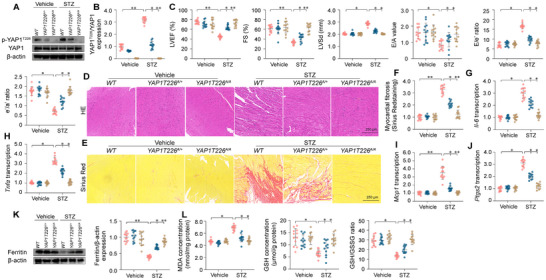
Repression of YAP Thr226 phosphorylation attenuates hyperglycemia‐mediated heart dysfunction. Heterozygous *YAP1T226^A/+^* mice, homozygous *YAP1T226^A/A^* mice, and wild‐type (WT) mice (8–10 weeks of age) were injected intraperitoneally with streptozotocin (STZ, 50 mg kg^−1^ in 0.1 mol L^−1^ citrate buffer) for five consecutive days to induce diabetes. Age‐ and sex‐matched non‐diabetic control mice were injected with an equal volume of PBS. A,B) Western blot analysis of phosphorylated YAP1 at Thr226 (p‐YAP1^T226^) and total YAP1 expression in WT, *YAP1T226^A/+^*, and *YAP1T226^A/A^* mice treated with PBS or STZ. C) Echocardiographic analysis of cardiac function. D) Representative histopathological images (H&E staining) showing myocardial disarray in diabetic mice. E) Representative histopathological images (Sirius Red staining) illustrating myocardial fibrosis in diabetic mice. F) Quantification of Sirius Red staining to assess myocardial fibrosis. G–J) Quantitative PCR (qPCR) analysis of *Il‐6, Tnfα*, *Mcp1*, and *Ptgs2* mRNA expression in heart tissues. K) Western blot analysis of ferritin expression in heart tissue extracts. L) ELISA‐based quantification of malondialdehyde (MDA), glutathione (GSH), and the GSH/GSSG ratio in heart tissues. Each group consisted of 4 animals or 4 independent cell culture experiments (*n* = 4). For each animal or independent cell culture experiment, measurements were repeated three times under the same experimental conditions. In each panel, dots represent individual measurements from animals or independent cell culture experiments. Bars represent group means, and error bars indicate ± standard error (SD). *p < 0.05, **p < 0.01, ***p < 0.001, ns, not significant.

To investigate the role of YAP1 phosphorylation in DCM, we generated a genetically modified mouse line with a YAP1 knock‐in mutation on a C57BL/6 background, substituting alanine for threonine at position 226 (T226A). Both heterozygous *YAP1T226^A/+^* and homozygous *YAP1T226^A/A^* mice were viable and fertile, displaying normal growth and development without any observable anatomical, behavioral, or organ‐specific abnormalities, including in the liver, brain, and kidneys (Figure S10A–J, Supporting Information). Additionally, no significant differences were observed in heart function (**Figure** **9**A–C), myocardial fibrosis (Figure 9D–F), inflammatory response (Figure 9G–I), or ferroptosis (Figure 9J–L) among wild‐type (WT), heterozygous *YAP1T226^A/+^* and homozygous *YAP1T226^A/A^* mice.

However, notable changes became evident following STZ treatment. Phosphorylation of YAP1 at Thr226 was markedly increased in the hearts of WT mice, partially reduced in *YAP1T226^A/+^* mice, and completely absent in *YAP1T226^A/A^* mice (Figure 9A,B). Consistent with these observations, STZ‐induced myocardial dysfunction (Figure 9C), cardiac fibrosis (Figure 9D–F), pathological inflammation (Figure 9G–I), and ferroptosis (Figure 9J–L) were partially alleviated in *YAP1T226^A/+^* and significantly normalized in *YAP1T226^A/A^* mice. These findings indicate that YAP1 phosphorylation at Thr226 is critical for the development of hyperglycemia‐induced diabetic cardiomyopathy.

To further investigate the involvement of the DNA‐PKcs/YAP1 pathway in hyperglycemia‐induced myocardial injury, wild‐type (WT), *YAP1T226^A/+^* and *YAP1T226^A/A^* mice were treated with NU7441, a DNA‐PKcs inhibitor. Compared to non‐diabetic WT mice, NU7441 treatment significantly improved cardiac function (Figure S11A–F, Supporting Information), attenuated inflammation response (Figure S11G–I, Supporting Information), reduced myocardial fibrosis (Figure S11J–L, Supporting Information), and suppressed the transcription of ferroptosis‐related genes (Figure S11M,N, Supporting Information), an effect similar to that observed in *YAP1T226^A/A^* mice. Notably, STZ treatment partially impaired cardiac function, promoted cardiac fibrosis, and suppressed the expression of ferroptosis‐related genes (Figure S11A–N, Supporting Information) in *YAP1T226^A/+^* mice; however, these pathological alterations were alleviated by NU7441. In contrast, in *YAP1T226^A/A^* mice, NU7441 failed to provide additional cardioprotective effects regarding heart function, inflammation response, myocardial fibrosis, and the transcription of ferroptosis‐associated genes (Figure S11A–N, Supporting Information). Collectively, these results underscore the functional significance of DNA‐PKcs‐dependent YAP1 phosphorylation in the pathogenesis of hyperglycemia‐induced myocardial injury.

## 3 Discussion

Leveraging advanced gene modification technologies and proteomic analyses, our study sheds light on the intricate interplay between DDR, ferroptosis induction, and cardiomyocyte dysfunction in the setting of DCM. The central findings of this study reveal that: (1) hyperglycemia triggers DDR, leading to the upregulation of DNA‐PKcs in diabetic hearts; (2) genetic ablation of *DNA‐PKcs* ameliorates hyperglycemia‐induced cardiac dysfunction, reduces myocardial fibrosis, and mitigates inflammation; (3) mechanistically, ferroptosis operates downstream of DNA‐PKcs activation, with its reactivation reversing the cardioprotective effects of *DNA‐PKcs* deletion; (4) proteomics and Co‐IP experiments indicated that DNA‐PKcs directly interacts with and phosphorylates YAP1, with phosphorylation of YAP1 at Thr226 promoting its nuclear retention; (5) nuclear YAP1 upregulates ferroptosis‐associated gene transcription, exacerbating cardiomyocyte injury; and (6) mice expressing the YAP1‐T226A mutation, which prevents YAP1 phosphorylation, exhibit significantly improved cardiac function under hyperglycemic conditions. Collectively, these findings position DNA‐PKcs‐mediated YAP1 phosphorylation and subsequent ferroptosis activation as critical drivers of DCM, unveiling potential therapeutic targets for intervention.

The DNA‐PKcs is a pivotal constituent of the phosphatidylinositol 3‐kinase related kinase family, possessing the capacity to phosphorylate in excess of 700 substrates. Functioning as the primary enzyme, DNA‐PKcs collaborates with the Ku80/Ku70 heterodimer to form the active DNA‐PK holoenzyme, thereby fulfilling essential roles within the cellular DDR. Upon the manifestation of DSBs within cells, DNA‐PKcs is swiftly mobilized to the damaged sites and activated, thereby dictating cellular outcomes such as DSBs non‐homologous end joining (NHEJ) repair, pathway selection, replication stress response, cell cycle checkpoints, telomeres length conservation, senescence, and autophagy. Recent research has unveiled the multifaceted roles DNA‐PKcs plays in metabolic diseases. In fact, escalated DNA damage, senescence, and persistent DNA damage signaling have been documented in both type 1 and type 2 diabetes. Relative to healthy subjects, patients with T2DM exhibit heightened levels of basal endogenous and oxidative DNA.^[^ ^27^ ^]^ Additionally, diabetes patients demonstrate greater susceptibility to hydrogen peroxide and doxorubicin, and a diminished capacity to repair DNA damage instigated by these agents compared to their healthy counterparts.^[^ ^27^ ^]^ Corroborating these findings, a wealth of evidence attests to an escalated degree of DNA damage in the blood cells of diabetic patients.^[^ ^28^ ^]^ Complementary data from a diabetic model employing db/db diabetic mice revealed an augmented nuclear γH2AX signal across all tissues compared to the background control.^[^ ^29^ ^]^ Concurrently, DNA damage markers were also amplified in diabetes patients, including those with diabetic complications such as nephropathy and restrictive lung disease (RLD).^[^ ^29^ ^]^ Notably, increased liver stiffness was intimately tied to heightened DNA damage markers.^[^ ^29^ ^]^ Moreover, the progression of nephropathy over a 4‐year span was forecasted by increased DNA damage.^[^ ^29^ ^]^ In the context of STZ‐induced type‐1 diabetes, DNA damage and the presence of atypical DNA comets in the liver, kidney, brain, testes and pancreas were notably amplified post single‐dose administration of streptozotocin.^[^ ^30^ ^]^ In alignment with these findings, we validated the occurrence of DDR in the diabetic heart, and established a negative correlation between the extent of DDR and cardiomyocyte viability and myocardial function. Collectively, these insights reveal DDR as a previously unrecognized indicator of diabetic complications. Crucially, DNA‐PKcs, but not Ku80, has been identified as the sentinel and executor of DDR, thereby contributing to myocardial damage induced by hyperglycemia. As such, the mitigation of DDR damage or the inhibition of DNA‐PKcs should be incorporated into the design and development of pharmacological approaches aimed at therapeutically intervening diabetic complications.

A substantial body of evidence has illuminated the role of DNA‐PKcs in metabolic diseases. The knockdown of DNA‐PKcs via siRNA or its inhibition by specific DNA‐PKcs inhibitors has been shown to attenuate free fatty acid (FFA)‐induced upregulations of sterol regulatory element binding protein 1 (SREBP1) mRNA and nuclear active SREBP1 protein expressions, as well as reducing FFA‐induced upregulation of fatty acid synthase (FAS) promoter transcriptional activity and lipid accumulation.^[^ ^31^ ^]^ The disruption of pancreatic and duodenal homeobox 1 (PDX‐1) in insulin‐producing beta cells is reported to be associated with the development of diabetes.^[^ ^32^ ^]^ This effect is believed to be mediated by DNA‐PK activation‐induced PDX‐1 phosphorylation on threonine, followed by PDX‐1 degradation by the proteasome and a subsequent decline in PDX‐1‐mediated gene expression, such as GLUT2 and glucokinase, which have been identified as early indicators of diabetes.^[^ ^32^ ^]^ Moreover, in aging skeletal muscle, increased DNA breaks coincide with the activation of DNA‐PKcs.^[^ ^33^ ^]^ Conversely, reducing DNA‐PK activity enhances AMPK activity, preventing weight gain, mitochondrial function decline, and physical fitness deterioration in middle‐aged mice, offering protection against type 2 diabetes.^[^ ^33^ ^]^ DNA‐PKcs activation has also been reported to be associated with the transcriptional upregulation of lipogenic genes via upstream stimulatory factor 1 (USF1).^[^ ^34^ ^]^ LC‐MS/MS profiling of cardiac lipid metabolism in STZ‐induced DCM^[^ ^35^ ^]^ revealed significant dyslipidemia, characterized by elevated cholesterol, triglycerides, and oxidized low‐density lipoprotein (ox‐LDL), reflecting enhanced hepatic lipogenesis due to insulin resistance and impaired lipolysis. STZ treatment also upregulated long‐chain acyl‐CoA synthetase 4 (ACSL4),^[^ ^36^ ^]^ promoting the accumulation of polyunsaturated fatty acids, such as arachidonic acid, further contributing to dyslipidemia. Consistent with these findings, we observed increased triglyceride and cholesterol levels in STZ‐treated mice. Cholesterol, essential for membrane integrity, is primarily regulated in the liver, and cardiac‐specific *DNA‐PKcs* deletion did not affect its metabolism. In contrast, triglycerides, an important energy source for cardiomyocytes, were reduced in the *DNA‐PKcs*‐deficient group under hyperglycemic conditions. This reduction can be attributed to two mechanisms: (1) *DNA‐PKcs* deletion reduces cardiomyocyte death, thereby enhancing cell viability and promoting triglyceride uptake, and (2) *DNA‐PKcs* deletion partially reverses hyperglycemia‐induced suppression of fatty acid metabolism‐related gene transcription. Furthermore, *DNA‐PKcs* deficiency did not impact insulin levels. Since hyperinsulinemia is considered a potential contributor to DCM, it is noteworthy that STZ injection, which induces pancreatic damage, led to a significant reduction in insulin levels, resulting in hypoinsulinemia in this model. Importantly, *DNA‐PKcs* deletion in cardiomyocytes did not improve pancreatic insulin secretion, suggesting that the cardioprotective effects of *DNA‐PKcs* deficiency are independent of pancreatic function and serum insulin levels.

Beyond its involvement in metabolic disease, the pathological impact of DNA‐PKcs on various types of cardiovascular ailments has also been elucidated. *DNA‐PKcs* deletion has been shown to alleviate sepsis‐induced myocardial dysfunction by preserving mitochondrial integrity.^[^ ^37^ ^]^ Furthermore, in endotoxemia‐induced microvascular disorder, DNA‐PKcs has been reported to phosphorylate cofilin2, leading to cytoskeleton disruption in endothelial cells, thereby disturbing microcirculatory function in the heart.^[^ ^38^ ^]^ In a mouse model of cardiac ischemia‐reperfusion injury, DNA‐PKcs expression was found to markedly increase upon reperfusion injury.^[^ ^12^ ^]^ The deletion of *DNA‐PKcs* was observed to prevent the protein degradation of Bax inhibitor‐1, leading to reduced mitochondrial fission, enhanced mitophagy, and ultimately the preservation of cardiomyocyte survival.^[^ ^12^ ^]^ The deletion of DNA‐PKcs in vascular smooth muscle cells has been reported to reduce the size of neointimal lesions 3 weeks post wire‐injury.^[^ ^39^ ^]^ In both human and experimental pulmonary arterial hypertension, DNA‐PKcs protein levels were significantly elevated, implicating its involvement in hypoxic pulmonary vascular remodeling through its impact on cellular cycle machinery.^[^ ^40^ ^]^ In an angiotensin II‐induced hypertension model, DNA‐PKcs was found to directly interact with dynamin‐related protein 1 (Drp1), inducing mitochondrial fragmentation and resulting in the dedifferentiation of vascular smooth muscle cells.^[^ ^41^ ^]^ These findings underscore the role of DNA‐PKcs in metabolic disorder‐mediated myocardial dysfunction. Hyperglycemia was found to stimulate the expression and activity of DNA‐PKcs, which directly interacts with Yes‐Associated Protein 1 (YAP1), inducing its phosphorylation at Thr226. Altogether, these findings position DNA‐PKcs at the nexus of metabolic diseases and cardiovascular disorders. Considering that DNA‐PKcs inhibitors have been utilized in several clinical trials for cancer treatment,^[^ ^42^ ^]^ it appears promising to explore the application of DNA‐PKcs inhibitors in the treatment of metabolic disorder‐related cardiac dysfunction.

YAP1, a key effector of the Hippo pathway, is renowned for its roles in controlling organ size, facilitating tissue regeneration, and driving tumorigenesis. Recent research has suggested that YAP1 may also play a pivotal role in metabolic processes, contributing to the onset of metabolic disorders, such as diabetes‐accelerated atherosclerosis,^[^ ^43^ ^]^ diabetic kidney disease,^[^ ^44^ ^]^ and diabetic retinopathy.^[^ ^45^ ^]^ While several sophisticated molecular mechanisms have been proposed to elucidate the influence of YAP1 activation on the progression of metabolic disorders, the nuclear accumulation of YAP1 stands as a critical step. Once localized in the nucleus, YAP1 governs the transcription and expression of genes related to diabetic complications. The Neuropilin‐2 (Nrp2)/PlexinA1 pathway, which contributes to the proliferation of vascular smooth muscle cells (VSMCs) in diabetes‐associated intimal hyperplasia, is under YAP1's control.^[^ ^46^ ^]^ YAP1 protein levels were found to be elevated in adipose tissue from individuals with type 2 diabetes. Notably, the ablation of YAP1 reduced the expression of fibrosis‐related genes in adipose tissue, leading to improved glucose tolerance in comparison to littermate controls upon HFD treatment.^[^ ^47^ ^]^ Furthermore, hyperglycemia‐induced nuclear translocation of endothelial YAP1 and subsequent transcriptional expression of cyclooxygenase‐2 (COX‐2) and microsomal prostaglandin E synthase‐1 (mPGES‐1) contributed to increased prostaglandin E2 (PGE2) levels and platelet hyperreactivity.^[^ ^48^ ^]^ In a pressure overload‐induced myocardial dysfunction model in diabetic mice, YAP1 was activated in cardiomyocytes in response to HFD consumption and contributed to cardiomyocyte dedifferentiation by controlling its downstream target, transcriptional enhanced associate domain (TEAD1), a transcription factor.^[^ ^49^ ^]^ In line with previous studies, our findings also confirmed that YAP1 phosphorylation at Thr226 is associated with its retention in the nucleus, where it augments the transcription of ferroptosis‐related genes.

YAP1 is extensively phosphorylated at multiple sites, which differentially affect its stability, subcellular localization, and transcriptional activity. Phosphorylation by LATS1/2 kinases at residues such as Ser127 promotes YAP1 binding to 14‐3‐3 proteins and cytoplasmic retention, inhibiting its nuclear accumulation and transcriptional coactivation.^[^ ^50^ ^]^ Phosphorylation of YAP1 at Ser397 or Ser381 creates a docking site for casein kinase 1 (CK1), which further phosphorylates adjacent residues, facilitating recognition by the SCF(β‐TRCP) E3 ligase complex and leading to proteasomal degradation.^[^ ^51^ ^]^ In cholangiocarcinoma, phosphorylation of YAP1 at tyrosine 357 by LCK promotes its nuclear localization and transcriptional activation, driving tumor progression.^[^ ^25^ ^]^ In Drosophila and mammalian cells, PRP4K phosphorylation of Yki/Yap at S61/S109/S164/S384 promotes nuclear export and suppresses transcriptional activity, inhibiting tissue overgrowth.^[^ ^52^ ^]^ These phosphorylation events fine‐tune YAP1's function, either promoting its cytoplasmic retention,^[^ ^52^ ^]^ targeting it for degradation, or enhancing its stability and transcriptional activity,^[^ ^25^ ^]^ thus providing context‐dependent regulation in development, tissue homeostasis, and tumorigenesis.

Previous studies^[^ ^36^, ^53^ ^]^ have identified DNA‐PKcs as a key driver of inflammation in septic cardiomyopathy, with nuclear YAP1 translocation activating proinflammatory transcriptional programs (e.g., IL‐6, TNFα) through interactions with TEAD and other cofactors, leading to immune cell infiltration. These findings suggest that the DNA‐PKcs/YAP1 axis may regulate inflammation in DCM. Although our data show a reduction in inflammatory mediators following *DNA‐PKcs* deletion and YAP1 dephosphorylation, further investigation is needed to explore immune cell populations and the role of nuclear YAP1 in immune activation under hyperglycemic conditions. YAP1 activation also regulates genes related to oxidative phosphorylation (*ATP5F1A*, *UQCRFS1*, *SDHA*) and calcium handling (*RyR2*, *SLC8A1*),^[^ ^54^ ^]^ suggesting a direct role in cardiomyocyte contraction. Moreover, our observation that *DNA‐PKcs* deletion partially normalized triglyceride levels indicates a role for this pathway in regulating cardiomyocyte metabolism, particularly in the context of fatty acid versus glucose utilization and mitochondrial energy output. This suggests an indirect role for DNA‐PKcs in modulating cardiomyocyte contraction via the control of energy metabolism. Collectively, these findings underscore the role of the DNA‐PKcs/YAP1 axis in mediating impaired contractility and diminished cellular metabolism in the diabetic heart.

Numerous studies have highlighted the roles of DNA‐PKcs^[^ ^55^ ^]^ and YAP1^[^ ^54^, ^56^ ^]^ in fibroblast activation and fibrosis. This mechanism likely underlies the anti‐fibrotic effects observed following *DNA‐PKcs* deletion and YAP1 dephosphorylation in our study. Furthermore, the proinflammatory effects of DNA‐PKcs and YAP1 are well‐documented.^[^ ^53^, ^57^ ^]^ Since inflammatory cells can indirectly promote collagen production by secreting cytokines and activating fibroblasts,^[^ ^58^ ^]^ the proinflammatory actions of DNA‐PKcs and YAP1 likely create a feedforward loop that exacerbates fibroblast differentiation and collagen accumulation.

YAP1 has been identified as a core regulator of ferroptosis, promoting the expression of key genes (e.g., *SLC7A11*, *ACSL4*, *GPX4*) involved in lipid peroxidation and iron homeostasis,^[^ ^59^ ^]^ modulating the threshold for iron‐dependent oxidative damage. Although direct evidence linking DNA‐PKcs to ferroptosis is limited, its central roles in DNA repair and oxidative stress responses suggest it may indirectly influence cellular susceptibility to ferroptotic cell death. Our study further demonstrates that YAP1 acts as a downstream effector of DNA‐PKcs, providing a mechanistic explanation for the role of DNA‐PKcs in ferroptosis regulation.

Several limitations exist in this study. Although we demonstrate that DNA‐PKcs regulates inflammatory factors in cardiomyocytes during DCM, the contribution of infiltrating inflammatory cells to hyperglycemia‐induced myocardial inflammation cannot be excluded. Additionally, the origin of hyperglycemia‐induced myocardial fibrosis, whether from cardiac fibroblasts, inflammatory cells, or both, remains unclear. Previous studies suggest that collagen and other extracellular matrix (ECM) components are primarily synthesized by cardiac fibroblasts,^[^ ^60^ ^]^ while inflammatory cells promote collagen production by secreting cytokines (e.g., TGF‐β, TNF‐α, IL‐1β, IL‐6) and activating fibroblasts.^[^ ^58^ ^]^ In DCM, myocardial collagen is mainly secreted by fibroblasts, with inflammatory cells facilitating fibroblast activation through paracrine signaling or intercellular interactions. Our findings show that cardiomyocyte‐specific deletion of *DNA‐PKcs* reduces collagen deposition, suggesting that hyperglycemia‐induced fibrosis is likely driven by cardiomyocyte injury, apoptosis, and increased inflammatory mediators, which activate fibroblasts and attract inflammatory cells.

## 4 Conclusion

In conclusion, in the context of chronic hyperglycemia, a DDR is initiated, leading to the pathological activation of DNA‐PKcs in the diabetic heart. This activated DNA‐PKcs directly interacts with and phosphorylates YAP1 at Thr226, thereby increasing the nuclear expression of YAP1. Once in the nucleus, YAP1 enhances the transcription of genes associated with ferroptosis, engendering cardiomyocyte dysfunction. Our research delineates a regulatory step for ferroptosis activation via DNA‐PKcs‐mediated YAP1 phosphorylation and nuclear retention in diabetic cardiomyopathy. It provides insights into how inhibiting the DNA‐PKcs/YAP1/ferroptosis pathway could prove a valuable therapeutic strategy for treating hyperglycemia‐related myocardial dysfunction.

## 5 Experimental Section

### Animals

All procedures and animal experiments were executed in strict compliance with the Guidelines for the Care and Use of Laboratory Animals, as outlined by the US National Institutes of Health. The use of the animals was approved by the Animal Care and Ethics Committee of the Guangzhou University of Chinese Medicine (NO: 20211207002). Mice were bred under a 12/12‐h light/dark cycle, with unrestricted access to food and water. Throughout the experimentation and subsequent data analysis, the investigators remained blind to the mice genotypes. The cardiac‐specific conditional knockout line of DNA‐PKcs mice (*DNA‐PKcs^Cko^)* was established by crossbreeding *DNA‐PKcs^f/f^* mice with *α‐MHC* (alpha myosin heavy chain)‐*Cre* transgenic mice. Age‐matched *DNA‐PKcs^f/f^* mice were utilized as controls for *DNA‐PKcs^Cko^* mice. The *YAP1^T226A^* mutant mice were produced on a C57BL/6J genetic background (sourced from GemPharmatech, Jiangsu, China). The YAP1 gene was modified using CRISPR/Cas9 technology, and sgRNA and donor vectors were constructed in vitro. Cas9, sgRNA, and donor were then introduced into the fertilized eggs of C57BL/6J mice for homologous recombination. The resultant positive F0 mice were identified via PCR and sequencing analysis. Subsequently, a stable, inheritable F1 mice model was achieved by mating F0 mice with C57BL/6J mice, leading to the YAP1‐T226A point mutation on exon 13, where the 226th amino acid was mutated from threonine (T) to alanine (A). For the *YAP1^T226A^* mice experiments, wild type (WT) littermates were employed as controls. Diabetic cardiomyopathy was induced in 8‐week‐old male mice through a series of five consecutive days of intraperitoneal injections with STZ (Sigma; 50 mg kg^−1^ dissolved in 0.1 mol L^−1^ of citrate buffer). Following a period of 1 week post the final STZ injection, mice exhibiting fasting blood glucose levels ≥16.7 mM were classified as diabetic and chosen for experimentation.^[^ ^61^ ^]^ Age‐ and sex‐matched mice, treated with equivalent volumes of citrate buffer, served as non‐diabetic controls. All of the mice continued to be fed for 24 weeks, and blood glucose and body weight were regularly checked (Figure S1, Supporting Information). To activate DNA‐PKcs or ferroptosis, diabetic mice were treated with low dose of AsiDNA (3 mg kg^−1^) or RSL3 (10 mg kg^−1^) for 24 weeks, respectively. To inhibit the activity of DNA‐PKcs, diabetic mice were intraperitoneally injected with NU7441 (2 mg kg^−1^, Selleck, Catalogue: S7409) for 24 weeks.

### Metabolic Parameters Assessment

After a fasting period of 16 h, we conducted an intraperitoneal glucose tolerance test (IPGTT). This involved administering a glucose injection (Sigma, Cat# G7528) at a concentration of 2 g kg^−1^ body weight, diluted in sterile saline.^[^ ^62^ ^]^ We measured tail blood glucose levels (mmol L^−1^) using an AlphaTRAK glucometer at various intervals: prior to glucose administration (0 min), and then at 15, 30, 60, and 120 min post‐administration. Notably, anesthesia was deemed unnecessary for this procedure, and the mice were promptly returned to their original housing post‐test. For the cholesterol assessment, we employed a mouse total cholesterol ELISA kit (ab285242, Abcam), while the triglyceride levels were ascertained using an All Triglyceride Assay Kit (MBS3005147, MyBioSource, Inc.). These measurements were performed strictly adhering to the manufacturer's guidelines.

### Echocardiography Measurements

To meticulously scrutinize the modifications in cardiac function, we executed echocardiographic studies on both wild‐type and knockout mice.^[^ ^63^ ^]^ Following the precise measurement of the animals' weight, anesthesia was initiated with a dose of 3% isoflurane and subsequently sustained with 1.5% isoflurane throughout the echocardiographic examination. The subjects were delicately positioned in a supine position on a thermal pad to preserve their body temperature at a consistent 37 °C. The thoracic region was carefully sheared and swathed with a warm ultrasound‐conductive gel. Echocardiographic images were captured while cautiously maintaining the probe against the chest.

### Transmission Electron Microscopy

In line with the protocols previously delineated, we conducted a transmission electron microscopy examination of cardiac tissue samples. Initially, the preserved heart specimens were subjected to post‐fixation, dehydration, and subsequent embedding in a 100% resin medium. This was followed by precision cutting of 70‐nm ultra‐thin sections,^[^ ^64^ ^]^ which were then stained utilizing a 2% uranyl acetate solution. Subsequent to this, the sections were thoroughly rinsed with distilled water and treated with a lead‐stain solution to enhance contrast. The prepared grids were then scrutinized under a JEM‐1400Plus microscope (JEOL), allowing us to capture high‐resolution digital images with the aid of a VELETA camera (Olympus).

### Histology Assessment

Heart biopsy specimens were meticulously harvested and subsequently preserved in 1% formalin, followed by processing via standard clinical methodologies.^[^ ^65^ ^]^ Heart sections, embedded in paraffin and precisely sliced to a thickness of 4 µm, were treated with hematoxylin and eosin staining to facilitate routine evaluation. Observation was conducted using a Nikon Eclipse 80i microscope, a benchmark in optical technology. Fibrosis evaluation was performed employing both Masson Trichrome and Sirius Red staining techniques, recognized for their specific affinity to fibrotic tissue.

### Immunofluorescence

Fresh heart tissue specimens were immediately preserved in Optimal Cutting Temperature (OCT) compound at either −20 or −80 °C following collection. Subsequently, 4 µm thick sections were prepared from the frozen tissue samples.^[^ ^66^ ^]^ For the purpose of immunofluorescence staining, these frozen sections were subjected to fixation in a 10% formalin solution for a duration of 15 min at ambient temperature. This was followed by a series of three washes with Phosphate‐Buffered Saline with Tween 20 (PBST).^[^ ^64^ ^]^ To permeabilize the sections, they were treated with a 0.5% Triton X100 solution in PBS. In order to block non‐specific binding, sections were incubated with a 10% goat serum for a period of 1 h at room temperature. Primary antibodies were then introduced to the sections, which were left to incubate overnight at 4 °C. Upon completion of three subsequent washes, the sections were stained with corresponding fluorescence‐conjugated secondary antibodies. Nuclear staining was achieved through the application of 4′,6‐diamidino‐2‐phenylindole (DAPI). The imaging of these sections was carried out using a Zeiss LSM 510 microscope, and the specific primary antibodies employed in our study are detailed in Table‐S1, Supporting Information.

### Co‐Immunoprecipitation and Mass Spectrometry

In conducting the Co‐Immunoprecipitation (Co‐IP) assay, Protein A/G magnetic beads (sourced from Thermo Fisher Scientific, Rockford, Illinois, USA) underwent a pre‐coating process. This involved an incubation period with specific antibodies at a temperature of 4 °C over a duration of 2 h. The heart lysates were procured utilizing the Radio‐Immunoprecipitation Assay (RIPA) buffer, which contained both phosphatase and protease inhibitors, supplied by Beyotime.^[^ ^65^ ^]^ Following this, supernatants were gathered through centrifugation and subsequently incubated with the pre‐coated beads at a temperature of 4 °C, maintained throughout the night. The immunoprecipitated proteins attached to the beads were then collected and subjected to a washing procedure thrice using ice‐cold Phosphate‐Buffered Saline (PBS). Finally, the samples were analyzed either through Western blotting or submitted for Mass Spectrometry (MS) analysis. The specific antibodies employed in this process are detailed in Table S1, Supporting Information.

### Adult Mouse Cardiomyocyte Contraction Measurement

Adult murine left ventricular myocytes (AMLVMs) were isolated from diabetic mice utilizing the methodology delineated in previous work.^[^ ^67^ ^]^ The Ionoptix Calcium and Contractility System was employed to quantify cardiomyocyte contractions, using a stimulation pattern of 0.5 Hz square pulses (20 ms, 10 V) under ambient conditions.^[^ ^68^ ^]^ To accurately portray the mean values of the data analyzed, representative traces were meticulously chosen.

### Cell Culture and Treatment

HL‐1 cells were purchased from the ATCC. Cardiomyocytes cell lines were maintained in low‐glucose DMEM (Corning Cellgro, Corning, NY), supplemented with 10% FBS (Atlanta Biologicals, Atlanta, GA) and 1% penicillin/streptomycin (Invitrogen, Carlsbad, CA). HL‐1 cells were cultured under high glucose (HG, 30 mmol L^−1^) medium for 48 h to induce hyperglycemic stress. HL‐1 cells incubated in normal glucose (NG, 5.5 mmol L^−1^) medium were used as control. To inhibit DNA‐PKcs and Ku80 activities, cells were respectively treated with NU7441 (1 µM; Cat. No. S2638, Selleck Chemicals, Houston, TX, USA) and STL127705 (10 µM; Cat. No. HY122727, MedChemExpress, USA) for 2 h before high glucose treatment. AsiDNA (MW = 20 931.4 g mol^−1^) is a 64‐nucleotide (nt) oligodeoxyribonucleotide consisting of two 32 nt strands of complementary sequence connected through a 1.19bis(phospho)‐8‐hydraza‐2‐hydroxy‐4‐oxa‐9‐oxo‐nonadecane linker with cholesterol at the 5′‐end and three phosphorothioate internucleotide linkages at each of the 5′ and the 3′ ends. The sequence is: 5′‐XGsCsTsGTGCCCACAACCCAGCAAACAAGCCTAGALCLTCTAGGCTTGTTTGCTGGGTTGTGGGCACsAsGsC‐3′, where L is an amino linker, X a cholesteryl tetraethylene glycol, CL a carboxylic (hydroxyundecanoic) acid linker, and s is a phosphorothioate linkage. AsiDNA was synthesized and purified by LGC (UK) and kindly provided by Wael Jdey (Valerio Therapeutics). The stock concentration of AsiDNA dissolved in water was at 40 mg mL^−1^. To activate DNA‐PKcs, HL‐1 cells were treated with 5µM AsiDNA for 24 h before high glucose treatment.^[^ ^69^ ^]^ To induce ferroptosis, cells were treated with 10 µM RSL3 for 24 h before high glucose treatment.^[^ ^70^ ^]^

### Plasmid Construction and Transfection

Adenoviruses were produced utilizing the ViraPower Adenoviral Expression System (Invitrogen K493000), in strict accordance with the protocol provided by the manufacturer. We initiated the creation of stable, region‐specific DNA‐PKcs and YAP1 mutant constructs by generating cDNA from HL‐1 cells. The genes of interest were initially cloned into a pENTR2B vector (Invitrogen A10463), followed by subsequent cloning into a pAd/CMV/V5‐DEST Vector (Invitrogen V49320), facilitated by LR Clonase II Enzyme (Invitrogen 11791100) through in vitro recombination. We ensured the accuracy of all construct sequences through DNA sequencing. As a negative control, we utilized an empty vector that lacked the cDNA insert.^[^ ^71^ ^]^ For linearization, all constructs were transfected into the 293A cell line (Invitrogen R70507) using Lipofectamine 3000 Transfection Reagent (Invitrogen L3000001). Post‐transfection, the cells were re‐plated onto 10 cm dishes, culminating in the production of crude adenoviral lysate within an approximate timeframe of 10 days. This crude lysate was subsequently purified and amplified within 293A cells over a period of 24 to 48 h, resulting in the formation of the F1 virus. We determined the titer of the F1 adenoviruses using the Sea‐Plaque–agarose assay.^[^ ^72^ ^]^ Finally, to assess adenoviral infection efficiency into cardiomyocytes, we conducted tests using an optimal MOI, determined based on adenoviral‐GFP reporter infection efficiency. For transfection with Lipofectamine2000 (Thermo Fisher), cells were seeded into 6‐well plates coated with a minimal volume of 0.1% gelatin (Sigma). Transfection was done the following day for 4 h at 37 °C in DMEM supplemented with 3% FBS. OptiMEM was solely used for the formation of transfection complexes, using 5 µg RNA. Following transfection, the cells were washed twice with pre‐warmed PBS and the medium was replaced with DMEM supplemented with 10% FBS.

### RNA Interference and Transfection

Lentiviral‐based short hairpin RNAs (shRNAs) targeting mouse *FANCD2* (Santa Cruz Biotechnology, Cat. No. sc‐35357), *UBE2T* (Santa Cruz Biotechnology, Cat. No. sc‐106661), *DNA‐PKcs* (Santa Cruz Biotechnology, Cat. No. sc‐35201), or scramble shRNA targeting enhanced green fluorescent protein (GFP) (Santa Cruz Biotechnology, Cat. No. sc‐45924) were used for the knockdown experiments. To produce lentiviral particles, the shRNA lentiviral vectors were co‐transfected with the lentiviral packaging plasmids pLP1, pLP2, and pLP/VSVG (Invitrogen) into 293FT packaging cells using lipofectamine 2000 (Invitrogen) as the transfection reagent.^[^ ^73^ ^]^ At 72 h after transfection, the virus‐containing cell culture medium was harvested. A moderate multiplicity of infection (MOI = 3) was used for transduction of cells to minimize negative effects on cell proliferation.

### MTT, TUNEL, ELISA and Comet Assay

Cell viability was ascertained utilizing the MTT assay (DOJINDO), conforming to the guidelines provided by the manufacturer. Evaluation of cell death was performed on heart sections or cellular samples using the Dead End Fluorometric TUNEL System (Promega, #G3250) adhering to the manufacturer's protocol. The detection of dead cells was achieved through confocal microscopy visualization. Serum or cellular supernatant levels of cytokines and chemokines, namely MDA, GSH, GSH/GSSG, LDH, were gauged utilizing ELISA kits (NeoBioscience Technology, Shenzhen, China),^[^ ^74^ ^]^ in accordance with the manufacturer's instructions. DNA damage was evaluated via the comet assay kit (Trevigen, cat. no. 4250‐050K) under alkaline conditions, with mean tail moments quantified for 300–500 cells per sample using specialized software (Comet Assay Software Project v1.2.3b1).

### Western Blot Analyses

Cellular and tissue lysates were prepared in adherence to standardized protocols. For cell samples, lysates were obtained using T‐PER buffer (Fisher Scientific) complemented with Halt protease and phosphatase inhibitors (Thermo Fisher). Following preparation, the lysates were clarified via centrifugation at 10 000 g for a duration of 20 min at 4 °C. Post‐centrifugation, the supernatant was treated with loading buffer and denatured through a 5‐min boiling process. In parallel, mouse heart tissue lysates were prepared by homogenizing 75 mg of ground frozen tissue in RIPA buffer supplemented with an array of inhibitors.^[^ ^75^ ^]^ Centrifugation at 14 000 g for 30 min at 4 °C was utilized to clarify the tissue samples, followed by the addition of SDS‐PAGE loading buffer and denaturation via boiling for 5 min. Prior to loading onto SDS‐PAGE gels, the denatured lysate was briefly sonicated. Equal quantities of proteins (20 µg from cells, 50 µg from tissues) were loaded and resolved on 10% SDS‐PAGE gels. The protein bands were then transferred to a PVDF membrane (Millipore). The membranes were subsequently developed using horseradish peroxidase (HRP)‐conjugated secondary antibodies at a dilution ratio of 1:1000, and visualized using ECL reagent (Pierce, Rockford, IL). The primary antibodies utilized are listed in Table S1, Supporting Information. Signal intensities of both phosphorylated and total proteins were quantified using Image J software (NIH).

### Quantitative Real‐Time PCR

Total RNA was meticulously isolated from frozen heart homogenates or cellular specimens utilizing the RNeasy Mini kit (Qiagen). Subsequent to this, cDNA was synthesized via reverse transcriptase.^[^ ^76^ ^]^ We performed real‐time quantitative PCR for numerous gene targets employing the SYBR Green Master Mix (Roche), using a stringent 40‐thermocycling protocol.^[^ ^77^ ^]^ The data derived from the ΔΔCT method are presented as relative‐fold changes in mRNA, normalized to the specified housekeeping gene. Detailed information regarding the primers utilized in our study is provided in Table S2, Supporting Information.

### Ethics and Human Sample Procurement

This study was reviewed and approved by the local medical ethics committee of Guangzhou University of Chinese Medicine (NO: 20211207002). Prior to enrolment, comprehensive informed consents were obtained from all participating patients. Diagnosis of diabetic cardiomyopathy was made based on the 2023 ESC Guidelines for the management of cardiovascular disease in patients with diabetes.^[^ ^78^ ^]^ Accordingly, blood specimens were sourced from both diabetic cardiomyopathy‐afflicted patients (*n* = 50) and those absent of the condition (*n* = 50). A detailed breakdown of patient demographics is presented in Table S3, Supporting Information.

### In Vitro Kinase Assay

The in vitro kinase assays were conducted employing His‐tagged recombinant mouse DNA‐PKcs protein (Catalogue No. LS‐G26073‐200, Nordic Biosite) and recombinant mouse YAP1 protein (Catalogue No. TP512130, Nordic Biosite).^[^ ^79^ ^]^ These assays were performed utilizing an Universal Kinase Activity Kit (Catalogue No. EA004, Bio‐Techne Corp., Minneapolis, MN, USA), strictly adhering to the manufacturer's guidelines.

### Molecular Docking and Dynamics Simulation

Investigations into the protein structures of DNA‐PKcs and YAP1 for docking studies were conducted utilizing the ZDOCK module from Discovery Studio 2019. Specific parameters were meticulously designated: an Euler angle of 6°, a Top Poses parameter of 2000, an RMSD Poses parameter of 1.0 nm, and a Maximum Number of Clusters parameter set at 100. All other parameters were left at their default settings. This led to the creation of a preliminary DNA‐PKcs/YAP1 conformation, which served as an input structure for subsequent molecular dynamics simulations via Gromacs 2019. The resulting stable binding mode between DNA‐PKcs and YAP1 was duly noted. The binding free energies of the DNA‐PKcs/YAP1 complex were calculated using the Calculate Binding Energies method, a feature native to Discovery Studio. To pinpoint the stable conformation for the dynamics simulation of DNA‐PKcs/YAP1, a virtual saturation mutation was executed.^[^ ^80^ ^]^ This involved mutating the interaction interface residues of DNA‐PKcs to each essential amino acid individually, followed by a comprehensive analysis of mutation energy. Moreover, the interaction interface residues of DNA‐PKcs were subjected to virtual alanine scanning, consistent with the Build Mutation Protocol of the Protein Design module in Discovery Studio 2019. The binding energy was then calculated using the Calculate Binding Energy Protocol of Discovery Studio 2019. Finally, high‐resolution molecular graphics representing the optimal binding pose were generated using PyMOL (DeLano Scientific) and LigPlus, providing a detailed visual representation of the binding process.^[^ ^81^ ^]^

### DNA Damage (8‐hydroxydeoxyguanosine) Assay

The quantification of 8‐OHdG was carried out utilizing the Cloud‐Clone Crop 8‐OHdG Competitive Inhibition Enzyme Immunoassay Kit (Cloud‐Clone Crop., USCN Life Science Inc., Houston, TX, USA).^[^ ^82^ ^]^ This kit is designed for the quantitative assessment of 8‐OHdG in human serum. A microplate pre‐coated with a monoclonal antibody specific to 8‐OHdG was employed. Subsequently, a competitive inhibition reaction was initiated involving biotin‐labeled 8‐OHdG and unlabeled 8‐OHdG (standards or samples) with the pre‐coated antibody. Upon incubation, the unbound conjugate was thoroughly washed off. Following this, each microplate well was treated with avidin conjugated to horseradish peroxidase (HRP) and incubated for 30 min at 37 °C.^[^ ^83^ ^]^ Notably, the quantity of the HRP conjugate bound was inversely related to the 8‐OHdG concentration in the sample. Upon the addition of the substrate solution, absorbance was measured at a wavelength of 450 nm using a microplate reader. The developed color intensity was also found to be inversely proportional to the 8‐OHdG concentration. The serum 8‐OHdG levels were deduced from the standard curve, with results expressed in picograms per milliliter (pg mL^−1^).

### Statistical Analysis

Data are presented as the mean± standard deviation (SD). Statistical analyses were conducted using the Statistical Product and Service Solutions (SPSS, version 19.0) software. For data conforming to a normal distribution, comparisons between two groups were performed using a two‐tailed Student's *t*‐test, while One‐way Analysis of Variance (ANOVA) was employed for multiple group comparisons. Subsequent analyses were carried out with Bonferroni's test, contingent on the data meeting the assumption of variance homogeneity, or Tamhane's T2 test, in the case of heteroscedastic data. Each group consisted of 4 animals or 4 independent cell culture experiments (*n* = 4). For each animal or independent cell culture experiment, measurements were repeated three times under the same experimental conditions. In each panel, dots represent individual measurements from animals or independent cell culture experiments. Bars represent group means, and error bars indicate ± standard error (SD). *p < 0.05, **p < 0.01, ***p < 0.001, ns, not significant.

## Conflict of Interest

The authors declare no conflict of interest.

## Supporting information



Supporting Information

## Data Availability

The data that support the findings of this study are available from the corresponding author upon reasonable request.
