# Supplementary material for: DNA‐PKcs‐Driven YAP1 Phosphorylation and Nuclear Translocation: a Key Regulator of Ferroptosis in Hyperglycemia‐Induced Cardiac Dysfunction in Type 1 Diabetes
Source: Adv Sci (Weinh). 2025 Apr 25;12(28):2412698. doi: 10.1002/advs.202412698 (PMC12302537; doi:10.1002/advs.202412698)

**Supporting Information**

**DNA-PKcs-Driven YAP1 Phosphorylation and Nuclear Translocation: A Key Regulator of Ferroptosis in Hyperglycemia-Induced Cardiac Dysfunction in Type 1 Diabetes**

**Table S1. Antibody information**

| Name | Catalogue number | Dilution factor |
| --- | --- | --- |
| DNA-PKcs | Abcam, #ab32566 | 1:1000 |
| p-DNA-PKcs | Abcam, #ab18192 | 1:1000 |
| γH2AX | Abcam, #ab81299 | 1:1000 |
| MDC1 | Abcam, #ab271061 | 1:1000 |
| 53BP1 | Abcam, #ab175933 | 1:1000 |
| β-actin | Abcam, #ab8226 | 1:1000 |
| Ku80 | Abcam, #ab80592 | 1:1000 |
| ATM | Abcam, #ab32420 | 1:1000 |
| p-ATM | Abcam, #ab81292 | 1:1000 |
| Collagen I | Abcam, #ab138492 | 1:1000 |
| SLC7A11 | Abcam, #ab307601 | 1:1000 |
| Histone H3 | Abcam, #ab308373 | 1:1000 |
| Collagen III | Abcam, #ab184993 | 1:1000 |
| TnT | Abcam, #ab8295 | 1:1000 |
| TGFβ | Abcam, #ab215715 | 1:1000 |
| Ferritin | Abcam, #ab75973 | 1:1000 |
| YAP1 | Abcam, #ab205270 | 1:1000 |
| LATS2 | Abcam, #ab243657 | 1:1000 |
| MST1 | Abcam, #ab245190 | 1:1000 |

**Table S2. Primers for qPCR**

| Gene | Forward Primer | Reverse Primer | |
| --- | --- | --- | --- |
| Mouse *Il-6* | 5'-CAGACTCGCGCCTCTAAGGAGT-3' | 5'-GATAGCCGATCCGTCGAA-3' |  |
| Mouse *Mcp1* | 5'-GATAGCCGATCCGTCGAA-3' | 5′-GCTACCACAACATCTGGACATT-3′ |  |
| Mouse *Mmp9* | 5′-AACCAATGATGCTGGGTTCAC-3′ | 5'-GCGCCGACTCAGAGGTGT-3' |  |
| Mouse *Ptgs2* | 5′-TTCCTCCTGTGCCTGATGATT-3′ | 5′-AAACTGATGCGTGAAGTGCTG-3′ |  |
| Mouse  *β-actin* | 5′-GGCTGTATTCCCCTCCATCG-3′ | 5′-CCAGTTGGTAACAATGCCATGT-3′ |  |
| Mouse *Slc1a5* | 5′-TGGAGATGAAAGACGTCCGC-3′ | 5′-CAGGCAGGCTGACACTGGAT-3′ |  |
| Mouse  *Cpt1a* | 5′-GGTCTTCTCGGGTCGAAAGC-3′ | 5′-TCCTCCCACCAGTCACTCAC-3′ |  |
| Mouse *Cpt2* | 5′-CAACTCGTATACCCAAACCCAGTC-3′ | 5′-GTTCCCATCTTGATCGAGGACATC-3′ |  |
| Mouse *Pparα* | 5′-AGAAGTTGCAGGAGGGGATT-3′ | 5′-TTGAAGGAGCTTTGGGAAGA-3′ |  |

**Table S3. Demographics of patients diagnosed with (+) or without (-) diabetic cardiomyopathy (DCM)**

| Patient characteristics | | DCM (-) patients (n=50) | DCM (+) patients (n=50) |
| --- | --- | --- | --- |
| Age | | 51.5±4.7 | 59.1±2.9 |
| Sex | | Male (n=31) | Male (n=39) |
| Body weight (kg) | | 71.36±2.74 | 84.34±2.98 |
| Comorbidities (n) | |  |  |
| Atrial fibrillation | 2 | 42 |  |
| Heart failure | 2 | 46 |  |
| Hypertension | 6 | 48 |  |
| Tumor | 1 | 2 |  |
| Coronary artery disease | 11 | 47 |  |
| Echocardiography | |  |  |
| LVEF (%) | | 68.36±3.47 | 53.19±3.43 |
| E/A | | 1.55±0.15 | 1.16±0.06 |
| E/e’ | | 7.83±0.98 | 14.20±0.83 |
| FS (%) | | 50.74±8.3 | 41.26±9.2 |
| Fasting Blood Sugar (mg/dL) | | 89.78±1.81 | 125.52±4.24 |
| Postprandial 2h Blood Sugar (mg/dL) | | 123.06±2.53 | 185.46±5.43 |
| HbA1c (%) | | 5.35±0.11 | 7.66±0.17 |

**Figure S1. Schematic of the experimental design for the mouse study.**

Diabetic cardiomyopathy was induced in eight-week-old male mice through five consecutive days of intraperitoneal injections with streptozotocin (STZ, 50 mg/kg dissolved in 0.1 mol/L citrate buffer). One week after the final STZ injection, mice with fasting blood glucose levels ≥16.7 mM were classified as diabetic and selected for further experimentation. Age- and sex-matched mice treated with equivalent volumes of citrate buffer served as non-diabetic controls. All mice were monitored for 24 weeks, with regular checks of blood glucose levels and body weight.


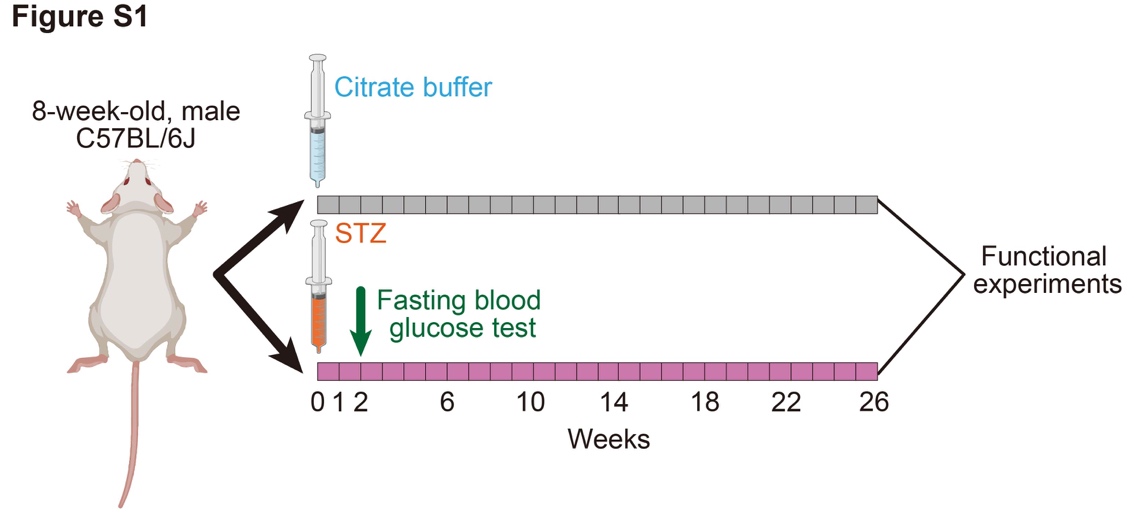


**Figure S2. DNA damage response (DDR) is activated in the hearts of STZ-injected rats.**

**(A-B)** Bioinformatics analysis performed using publicly available GSE197999 RNA-seq data from eight samples: four from STZ-induced diabetic cardiomyopathy in mouse hearts and four from control hearts.

**(C-E)** Gene Ontology (GO) analysis of differentially expressed genes.

**(F)** Kyoto Encyclopedia of Genes and Genomes (KEGG) pathway analysis of altered genes.

**(G)** Gene Set Enrichment Analysis (GSEA) plots illustrating the enrichment of DDR-related processes.

**
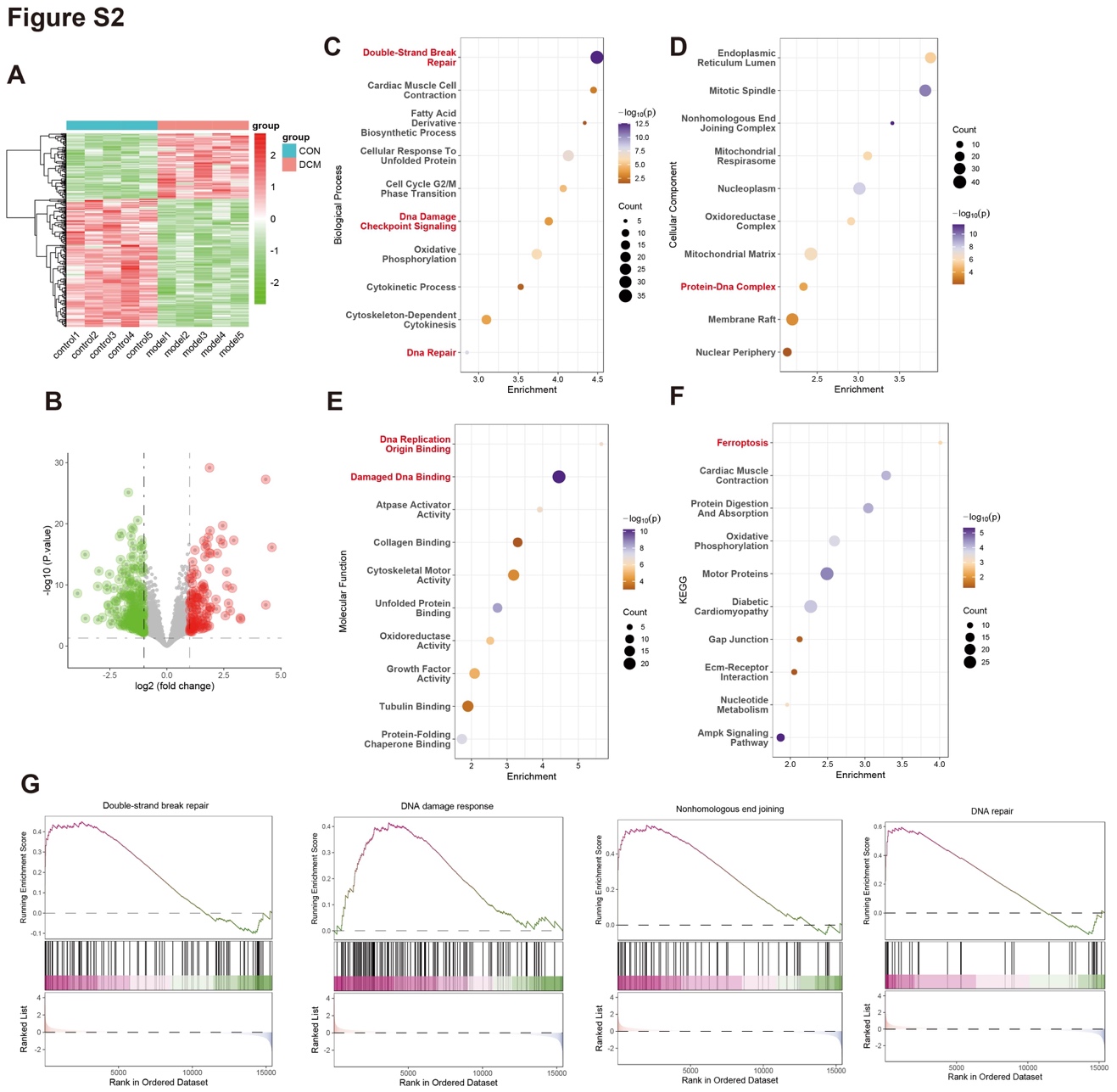
**

**Figure S3. DNA-PKcs regulates the DNA damage response (DDR) and contributes to hyperglycemia-induced cardiomyocyte dysfunction.**

In vitro, HL-1 cells were cultured in high glucose (HG, 30 mmol/L) medium for 48 hours to induce hyperglycemic stress, while cells incubated in normal glucose (NG, 5.5 mmol/L) medium served as controls.

**(A)** HL-1 cells were transduced with sh-scramble, sh-FANCD2, or sh-UBE2T, and cell viability was assessed using the MTT assay.

**(B)** ELISA was used to measure lactate dehydrogenase (LDH) levels in the culture media of HL-1 cells transduced with sh-scramble, sh-FANCD2, or sh-UBE2T under high glucose (30 mmol/L) conditions.

**(C-E)** HL-1 cells were transfected with Ad-FANCD2. Proteins were then isolated from HG- or NG-treated cells, and the expression of γH2AX, MDC1, and 53BP1 was assessed by Western blotting.

**(F-H)** HL-1 cells were transfected with Ad-UBE2T. Proteins were then isolated from HG- or NG-treated cells, and the expression of γH2AX, MDC1, and 53BP1 was determined by Western blotting.

**(I)** Cell viability in HL-1 cells transfected with Ad-FANCD2 was assessed using the MTT assay and LDH release assay.

**(J)** Cell viability in HL-1 cells transfected with Ad-UBE2T was assessed using the MTT assay and LDH release assay.

**(K)** MTT assay was used to evaluate cell viability in HL-1 cells infected with sh-Ku80. ELISA was used to measure LDH levels in the culture media of HL-1 cells infected with sh-Ku80.

**(L)** MTT assay was used to evaluate cell viability in HL-1 cells treated with the Ku80 inhibitor STL127705. ELISA was used to measure LDH levels in the culture media of HL-1 cells treated with STL127705.

**(M)** MTT assay was used to evaluate cell viability in HL-1 cells transfected with Ad-Ku80 under high glucose (30 mmol/L) conditions. ELISA was used to measure LDH levels in the culture media of HL-1 cells transduced with Ad-Ku80 under high glucose (30 mmol/L) conditions.

Each group consisted of 4 animals or 4 independent cell culture experiments (n = 4). For each animal or independent cell culture experiment, measurements were repeated three times under the same experimental conditions. In each panel, dots represent individual measurements from animals or independent cell culture experiments. Bars represent group means, and error bars indicate ± standard deviation (SD). *p < 0.05; **p < 0.01; ***p < 0.001; ns, not significant; Glu: Glucose.


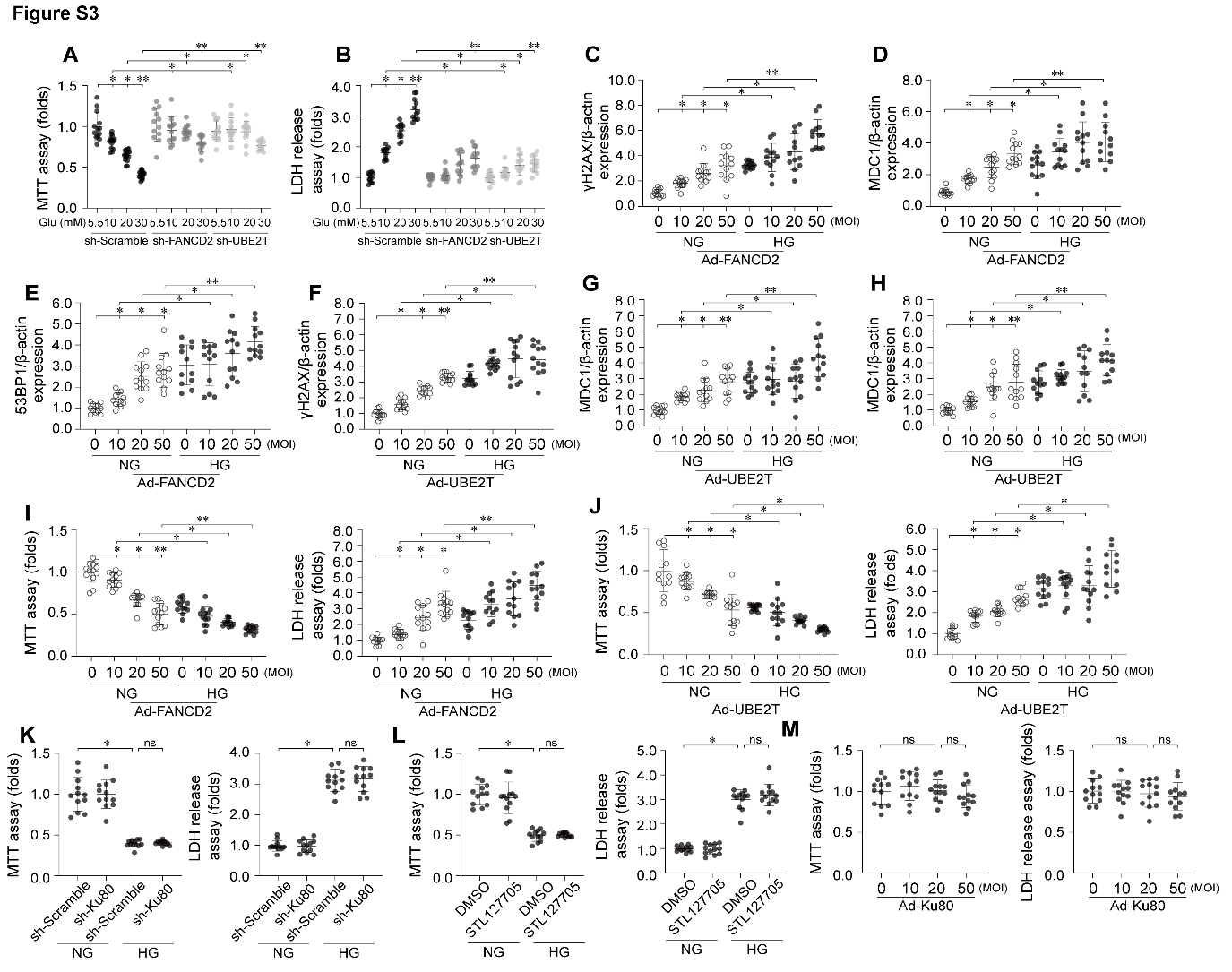


**Figure S4. Ablation of *Ku80* has no effect on systemic metabolic under hyperglycemic conditions.**

In vivo, cardiomyocyte-specific *Ku80* knockout (*Ku80^Cko^*) and wild-type *Ku80^f/f^* mice were injected intraperitoneally with streptozotocin (STZ, 50 mg/kg in 0.1 mol/L citrate buffer) for five consecutive days to induce diabetes. Age- and sex-matched non-diabetic mice were injected with an equal volume of PBS.

**(A)** Western blot analysis of phosphorylated DNA-PKcs (p-DNA-PKcs) in heart tissues from *DNA-PKcs^Cko^* and *DNA-PKcs^f/f^* mice. Each group consisted of 4 animals (n = 4). For each animal, measurements were repeated three times under the same experimental conditions. In each panel, dots represent individual measurements from animals. Bars represent group means, and error bars indicate ± standard error (SD). *p < 0.05; **p < 0.01; ***p < 0.001; ns, not significant.

**(B)** Western blot analysis of Ku80 protein expression in heart tissues from *Ku80^Cko^* and *Ku80^f/f^* mice. Each group consisted of 4 animals (n = 4). For each animal, measurements were repeated three times under the same experimental conditions. In each panel, dots represent individual measurements from animals. Bars represent group means, and error bars indicate ± standard error (SD). *p < 0.05; **p < 0.01; ***p < 0.001; ns, not significant.

**(C)** Time course of fasting blood glucose levels, measured at baseline (week 0) and periodically over the 24 weeks following STZ or vehicle (citrate buffer) injection. n=8 mice per group and dots in this panel represent the mean values derived from the data of these 8 mice.

**(D-E)** Fasting serum cholesterol (D) and triglyceride (E) levels in *Ku80^Cko^* and *Ku80^f/f^* mice. Each group consisted of 4 animals (n = 4). For each animal, measurements were repeated three times under the same experimental conditions. In each panel, dots represent individual measurements from animals. Bars represent group means, and error bars indicate ± standard error (SD). *p < 0.05; **p < 0.01; ***p < 0.001; ns, not significant.

**(F)** Time course of body weight changes. n=8 mice per group and dots in this panel represent the mean values derived from the data of these 8 mice.

**(G)** Intraperitoneal glucose tolerance test (IPGTT) performed 9 days after STZ or vehicle treatment in *Ku80^Cko^* and *Ku80^f/f^* mice. n=8 mice per group and dots in this panel represent the mean values derived from the data of these 8 mice.

**(H)** Levels of serum insulin in *DNA-PKcs^Cko^* and *DNA-PKcs^f/f^* mice. Each group consisted of 4 animals (n = 4). For each animal, measurements were repeated three times under the same experimental conditions. In each panel, dots represent individual measurements from animals. Bars represent group means, and error bars indicate ± standard error (SD). *p < 0.05; **p < 0.01; ***p < 0.001; ns, not significant.

**(I)** HL-1 cells were transduced with shRNA targeting DNA-PKcs (sh/DNA-PKcs) or with scramble control (sh/scramble), and then cultured in high-glucose (HG, 30 mmol/L) medium for 48 hours to mimic hyperglycemic stress. Cells incubated in normal glucose (NG, 5.5 mmol/L) medium served as controls. Then, RNA were isolated from cells and the qPCR assay was used to analyze the transcirption of *Cpt1a*, *Cpt2*, and *Ppar-α.* Each group consisted of independent cell culture experiments (n = 4). For each independent cell culture experiment, measurements were repeated three times under the same experimental conditions. In each panel, dots represent individual measurements from independent cell culture experiments. Bars represent group means, and error bars indicate ± standard error (SD). *p < 0.05; **p < 0.01; ***p < 0.001; ns, not significant.


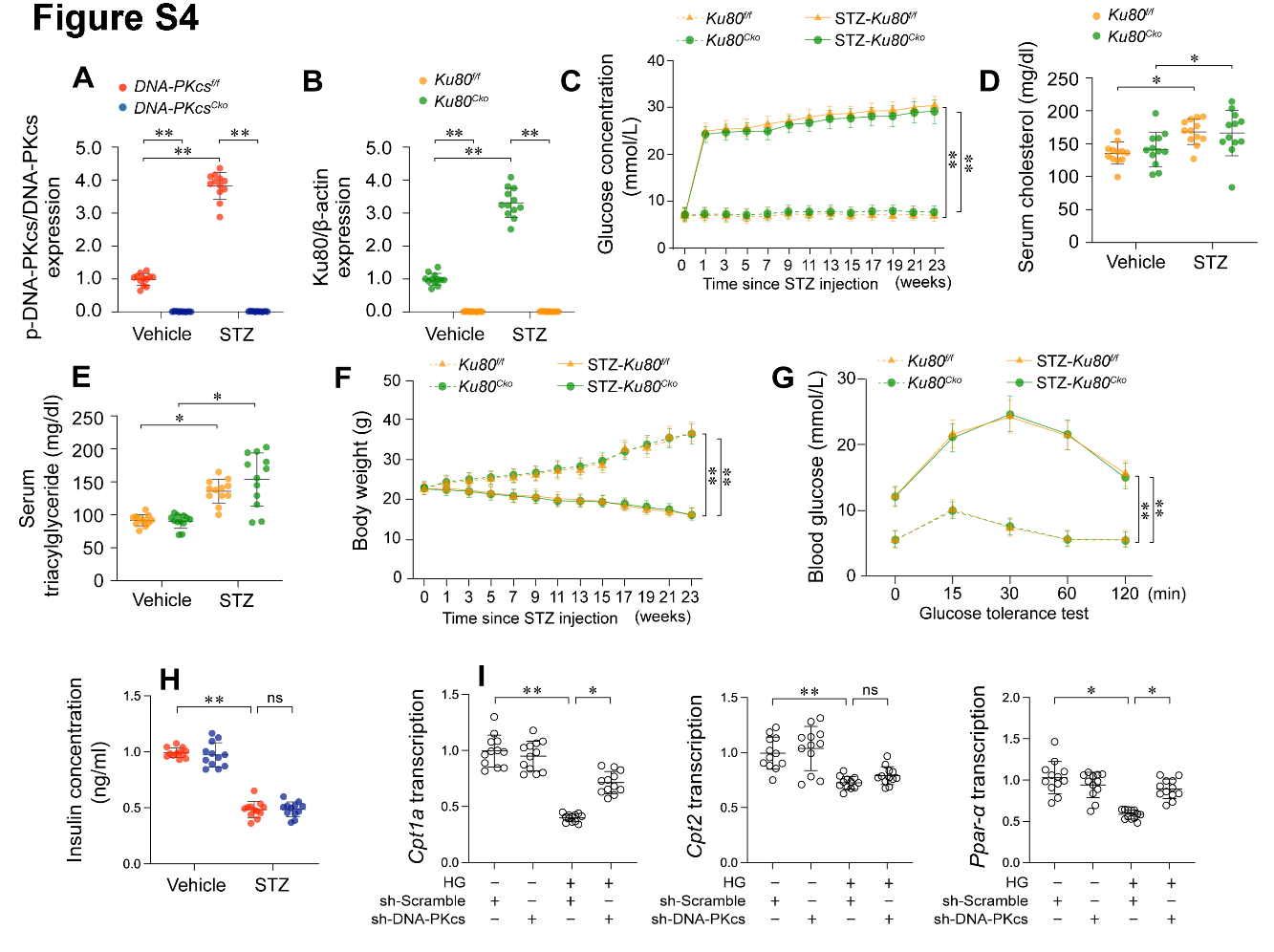


**Figure S5. Ablation of *Ku80* has no effect on heart function under hyperglycemic conditions.**

In vivo, cardiomyocyte-specific *Ku80* knockout (*Ku80^Cko^*) and wild-type *Ku80^f/f^* mice were injected intraperitoneally with streptozotocin (STZ, 50 mg/kg in 0.1 mol/L citrate buffer) for five consecutive days to induce diabetes. Age- and sex-matched non-diabetic mice were injected with an equal volume of PBS.

**(A)** Echocardiographic analysis of heart function, including left ventricular ejection fraction (LVEF), fractional shortening (FS), left ventricular systolic dimension (LVSd), left ventricular diastolic dimension (LVDd), early-to-late (atrial) mitral flow velocity ratio (E/A), ratio of mitral peak velocity of early filling to early diastolic mitral annular velocity (E/e’), and ratio of diastolic mitral annulus velocities (e’/a’).

**(B)** Analysis of contractile parameters in acutely isolated single cardiomyocytes from *Ku80^Cko^* and *Ku80^f/f^* mice, including peak shortening (PS), maximal velocity of shortening (+dL/dt), time-to-peak shortening (TPS), maximal velocity of relengthening (-dL/dt), and time-to-90% relengthening (TR90).

**(C)** Diabetic *Ku80^Cko^* and *Ku80^f/f^* mice were treated with a low dose of AsiDNA (3 mg/kg) for 24 weeks, followed by echocardiographic assessment of heart function.

Each group consisted of 4 animals or 4 independent cell culture experiments (n = 4). For each animal or independent cell culture experiment, measurements were repeated three times under the same experimental conditions. In each panel, dots represent individual measurements from animals or independent cell culture experiments. Bars represent group means, and error bars indicate ± standard deviation (SD). *p < 0.05; **p < 0.01; ***p < 0.001; ns, not significant.

**
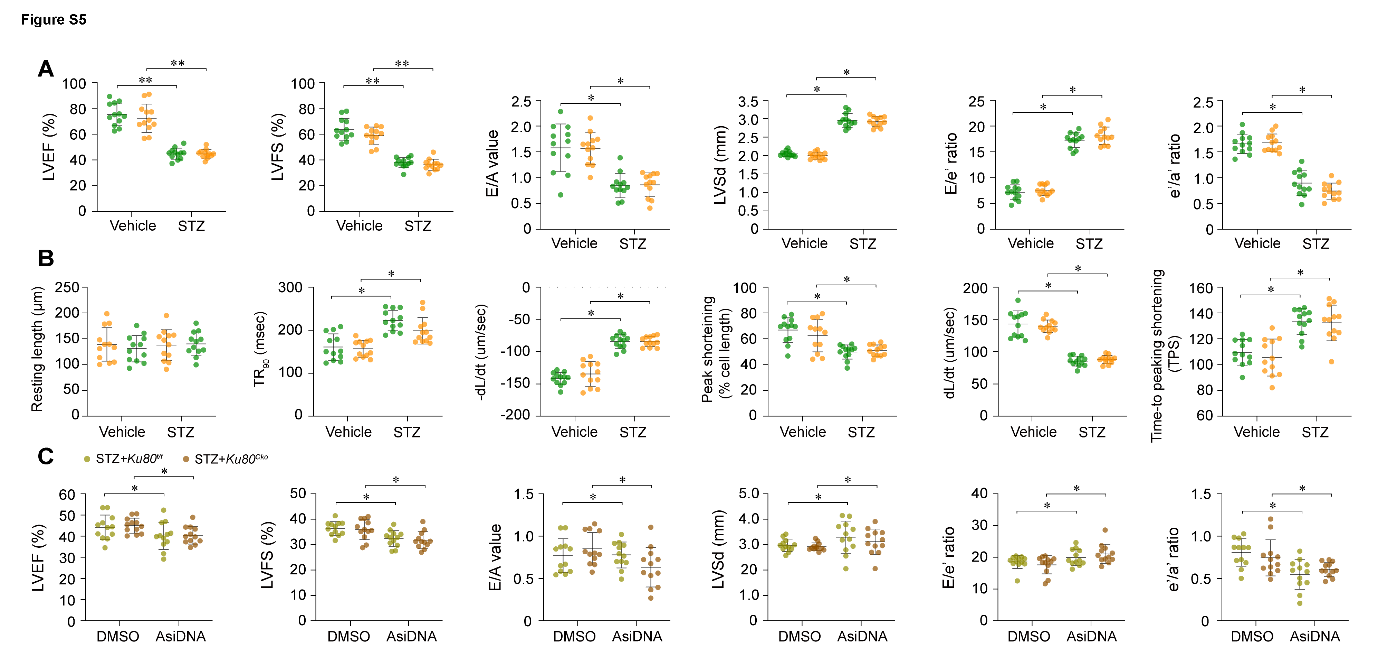
**

**Figure S6. *Ku80* ablation does not affect hyperglycemia-mediated myocardial structural disorder.**

In vivo, cardiomyocyte-specific *Ku80* knockout (*Ku80^Cko^*) and wild-type *Ku80^f/f^* mice were injected intraperitoneally with streptozotocin (STZ, 50 mg/kg in 0.1 mol/L citrate buffer) for five consecutive days to induce diabetes. Age- and sex-matched non-diabetic mice were injected with an equal volume of PBS.

**(A)** Representative histopathological images (H&E staining) showing myocardial disarray in diabetic mice.

**(B)** Representative histopathological images (Masson trichrome staining) revealing myocardial fibrosis in diabetic mice.

**(C)** Representative histopathological images (Sirius Red staining) illustrating myocardial fibrosis in diabetic mice.

**(D)** Quantification of myocardial fibrosis based on Masson trichrome staining.

**(E-G)** Western blot analysis of collagen I, collagen III, and TGFβ expression in heart tissue extracts.

**(H)** ELISA analysis of matrix metalloproteinase-9 (MMP9) activity in heart tissues.

**(I)** Quantitative PCR (qPCR) analysis of *Il-6*, *Tnfα*, and *Mcp1* mRNA expression in heart tissues.

**(J)** HL-1 cells were transduced with shRNA targeting DNA-PKcs (sh/DNA-PKcs) or with scramble control (sh/scramble), and then cultured in high-glucose (HG, 30 mmol/L) medium for 48 hours to mimic hyperglycemic stress. Cells incubated in normal glucose (NG, 5.5 mmol/L) medium served as controls. Quantitative PCR (qPCR) analysis of *Il-6*, *Tnfα*, and *Mcp1* mRNA expression in cardiomyocytes.

Each group consisted of 4 animals or 4 independent cell culture experiments (n = 4). For each animal or independent cell culture experiment, measurements were repeated three times under the same experimental conditions. In each panel, dots represent individual measurements from animals or independent cell culture experiments. Bars represent group means, and error bars indicate ± standard error (SD). *p < 0.05; **p < 0.01; ***p < 0.001; ns, not significant.


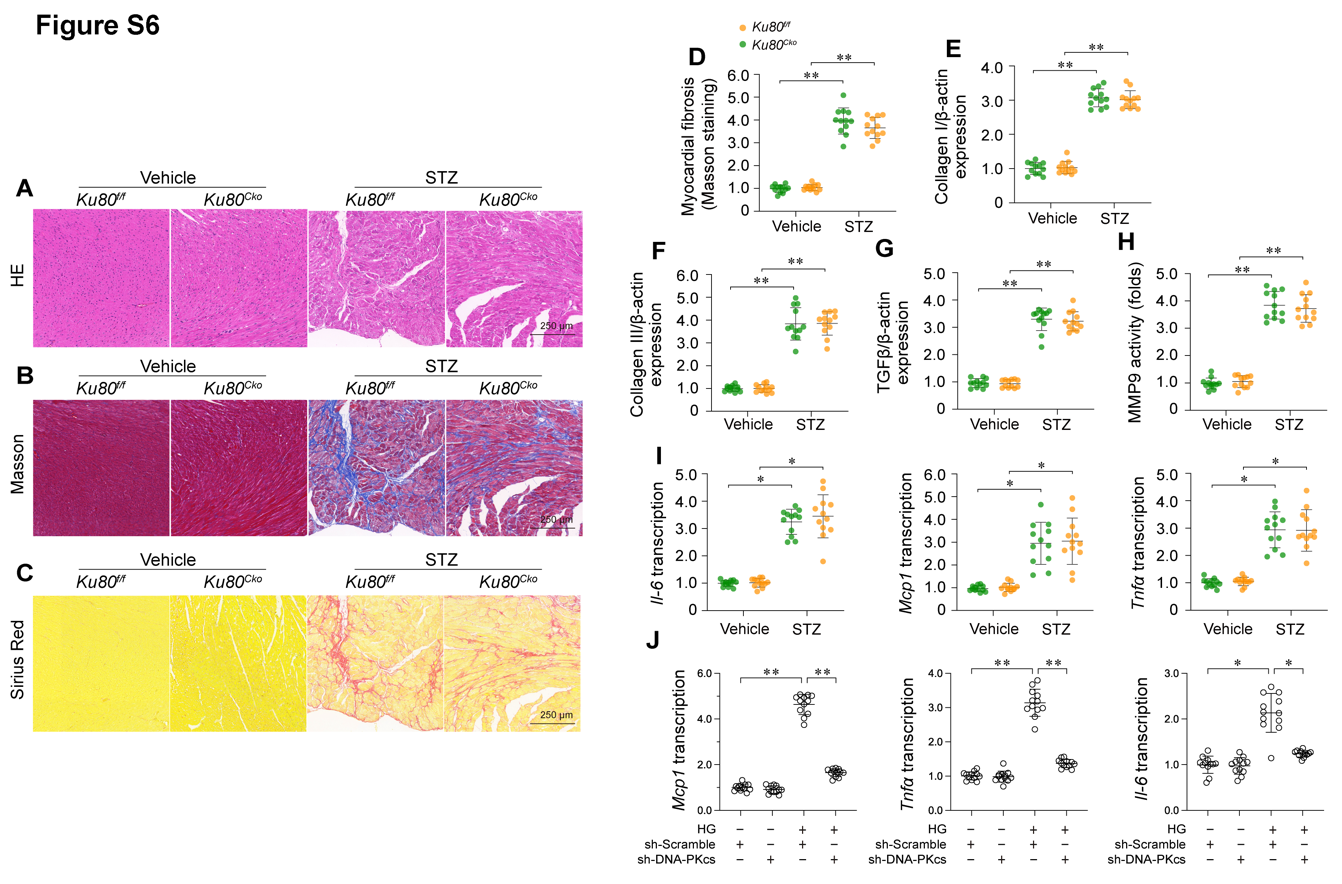


**Figure S7. DNA-PKcs does not interact with MST1 and LATS2.**

**(A)** Immunoprecipitates (IP) of DNA-PKcs or MST1 from heart tissues of mice with hyperglycemia-induced diabetic cardiomyopathy were immunoblotted (IB) as indicated.

**(B)** Immunoprecipitates of DNA-PKcs or LATS2 from heart tissues of mice with hyperglycemia-induced diabetic cardiomyopathy were immunoblotted as indicated.

**(C)** His-DNA-PKcs or HA-MST1 was transfected into HL-1 cells, followed by co-immunoprecipitation (Co-IP) assay.

**(D)** His-DNA-PKcs or HA-LATS2 was transfected into HL-1 cells, followed by Co-IP assay.

**(E)** HL-1 cells were treated with shRNA targeting MST1 (sh/MST1) before exposure to high glucose (HG), and qPCR analysis was used to assess Ptgs2 transcription.

**(F)** HL-1 cells were treated with sh/MST1 before HG exposure. Proteins were isolated, and ferritin expression was determined by Western blotting.

**(G)** HL-1 cells were treated with sh/MST1 before HG exposure, and ELISA kits were used to analyze the GSH/GSSG ratio.

**(H-I)** HL-1 cells were treated with sh/MST1 before HG exposure, and cell death was measured by MTT assay and LDH release assay.

**(J)** HL-1 cells were treated with shRNA targeting LATS2 (sh/LATS2) before HG exposure, and qPCR analysis was used to assess Ptgs2 transcription.

**(K)** HL-1 cells were treated with sh/LATS2 before HG exposure. Proteins were isolated, and ferritin expression was determined by Western blotting.

**(L)** HL-1 cells were treated with sh/LATS2 before HG exposure, and ELISA kits were used to analyze the GSH/GSSG ratio.

**(M-N)** HL-1 cells were treated with sh/LATS2 before HG exposure, and cell death was measured by MTT assay and LDH release assay.

Each group consisted of 4 animals or 4 independent cell culture experiments (n = 4). For each animal or independent cell culture experiment, measurements were repeated three times under the same experimental conditions. In each panel, dots represent individual measurements from animals or independent cell culture experiments. Bars represent group means, and error bars indicate ± standard error (SD). *p < 0.05; **p < 0.01; ***p < 0.001; ns, not significant.


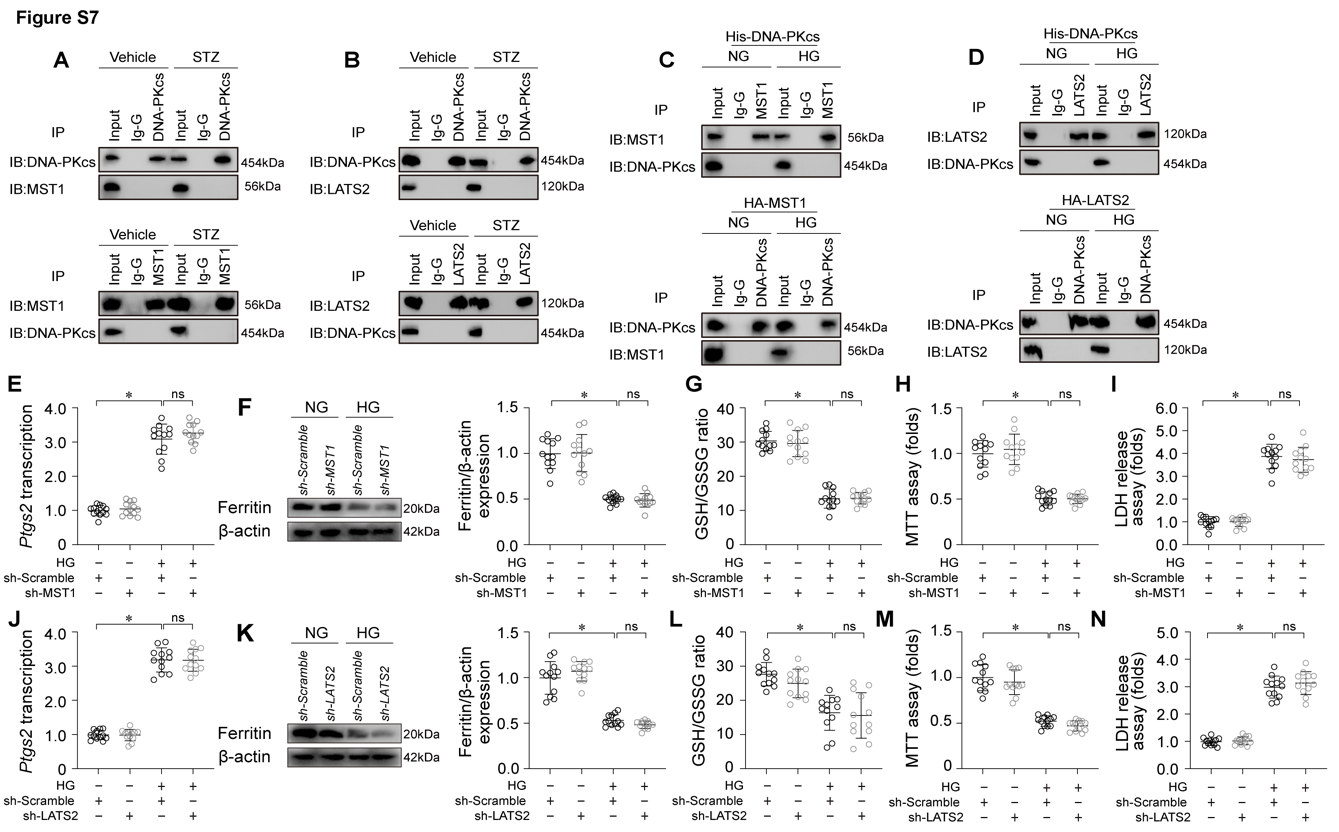


**Figure S8. The interaction between DNA-PKcs and YAP1 promotes ferroptosis.**

**(A)** HL-1 cells were transfected with various YAP1 constructs, including HA-YAP1ΔTEAD, HA-YAP1ΔWW, HA-YAP1ΔSH3, and HA-YAP1ΔTAD. qPCR analysis was then used to assess Ptgs2 transcription in HL-1 cells under HG exposure.

**(B)** HL-1 cells transfected with HA-YAP1ΔTEAD, HA-YAP1ΔWW, HA-YAP1ΔSH3, and HA-YAP1ΔTAD constructs were analyzed using ELISA kits to measure the GSH/GSSG ratio.

**(C-D)** Cell death in HL-1 cells transfected with HA-YAP1ΔTEAD, HA-YAP1ΔWW, HA-YAP1ΔSH3, and HA-YAP1ΔTAD constructs was measured by MTT assay and LDH release assay.

**(E)** HL-1 cells were transfected with various DNA-PKcs constructs, including His-DNA-PKcsΔN, His-DNA-PKcsΔFAT, and His-DNA-PKcsΔKinase. qPCR analysis was then performed to assess Ptgs2 transcription in HL-1 cells under high glucose (HG) exposure.

**(F)** HL-1 cells transfected with His-DNA-PKcsΔN, His-DNA-PKcsΔFAT, and His-DNA-PKcsΔKinase constructs were analyzed using ELISA kits to measure the GSH/GSSG ratio.

**(G-H)** Cell death in HL-1 cells transfected with His-DNA-PKcsΔN, His-DNA-PKcsΔFAT, and His-DNA-PKcsΔKinase was assessed using MTT assay and LDH release assay.

Each group consisted of 4 animals or 4 independent cell culture experiments (n = 4). For each animal or independent cell culture experiment, measurements were repeated three times under the same experimental conditions. In each panel, dots represent individual measurements from animals or independent cell culture experiments. Bars represent group means, and error bars indicate ± standard error (SD). *p < 0.05; **p < 0.01; ***p < 0.001; ns, not significant.


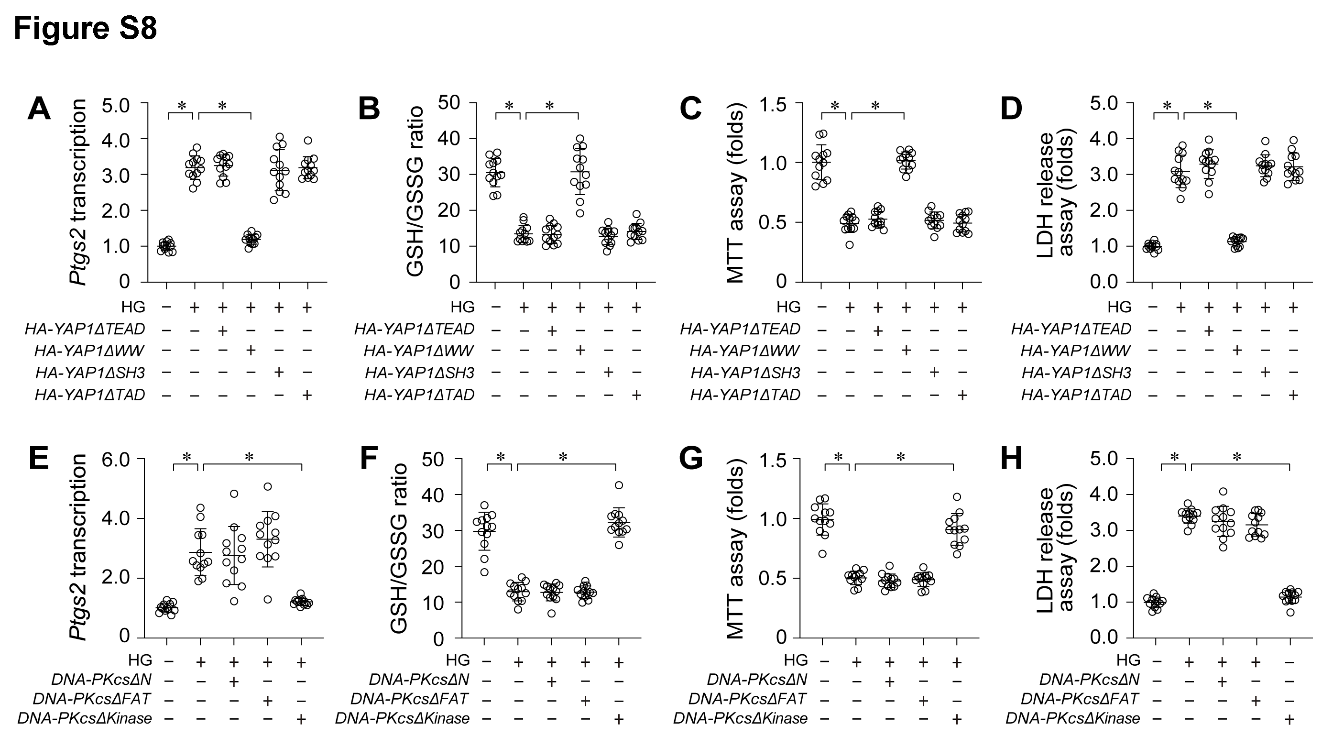


**Figure S9. YAP1 phosphorylation regulates its nuclear localization.**

**(A)** HL-1 cells were transfected with sh-DNA-PKcs, and YAP1 phosphorylation was assessed by Western blotting.

**(B)** Immunofluorescence was used to observe the nuclear localization of YAP1.

Each group consisted of 4 animals or 4 independent cell culture experiments (n = 4). For each animal or independent cell culture experiment, measurements were repeated three times under the same experimental conditions. In each panel, dots represent individual measurements from animals or independent cell culture experiments. Bars represent group means, and error bars indicate ± standard error (SD). *p < 0.05; **p < 0.01; ***p < 0.001; ns, not significant.


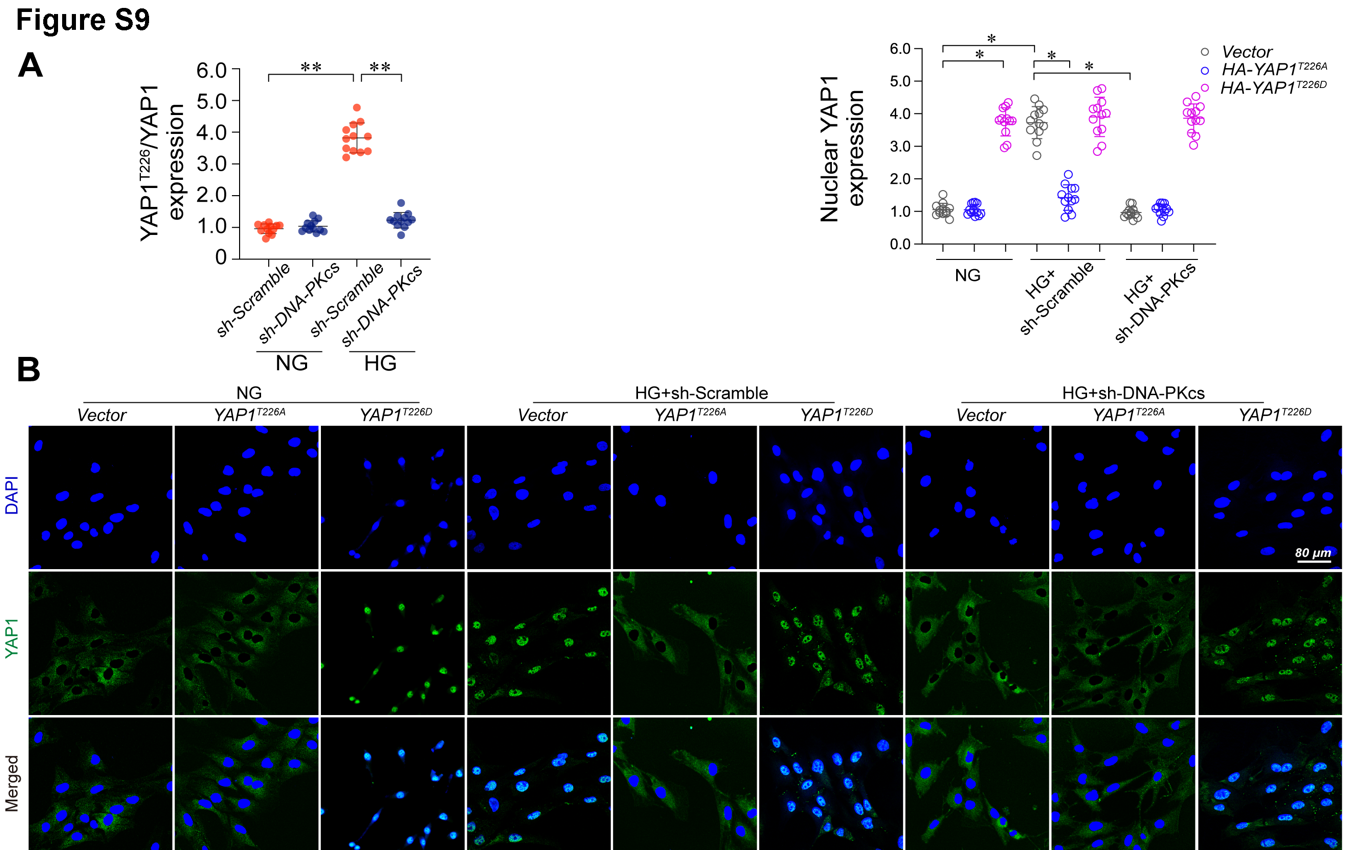


**Figure S10. A knockin mutation in *YAP1^T226A^* has no effect on liver, kidney, or brain structure and function.**

**(A-B)** Liver, kidney, and brain tissues were isolated from wild-type (WT), heterozygous *YAP1T226^A/+^* mice, and homozygous *YAP1T226^A/A^* mice. Histological analysis was performed using H&E staining, Sirius Red staining, TUNEL staining, and Nissl staining to assess structural changes in the liver, kidney, and brain.

**(C-E)** The levels of CK-MB, BNP, and Troponin T were measured by ELISA to evaluate cardiac function.

**(F-G)** ELISA analysis of blood urea nitrogen (BUN) and serum creatinine (Scr) concentrations in serum from WT, heterozygous *YAP1T226^A/+^*, and homozygous *YAP1T226^A/A^* mice.

**(H-I)** ELISA analysis of alanine transaminase (ALT) and aspartate transaminase (AST) concentrations in serum from WT, heterozygous *YAP1T226^A/+^*, and homozygous *YAP1T226^A/A^* mice.

**(J)** Survival rates of WT, heterozygous *YAP1T226^A/+^*, and homozygous *YAP1T226^A/A^* mice.

Each group consisted of 4 animals or 4 independent cell culture experiments (n = 4). For each animal or independent cell culture experiment, measurements were repeated three times under the same experimental conditions. In each panel, dots represent individual measurements from animals or independent cell culture experiments. Bars represent group means, and error bars indicate ± standard error (SD). *p < 0.05; **p < 0.01; ***p < 0.001; ns, not significant.


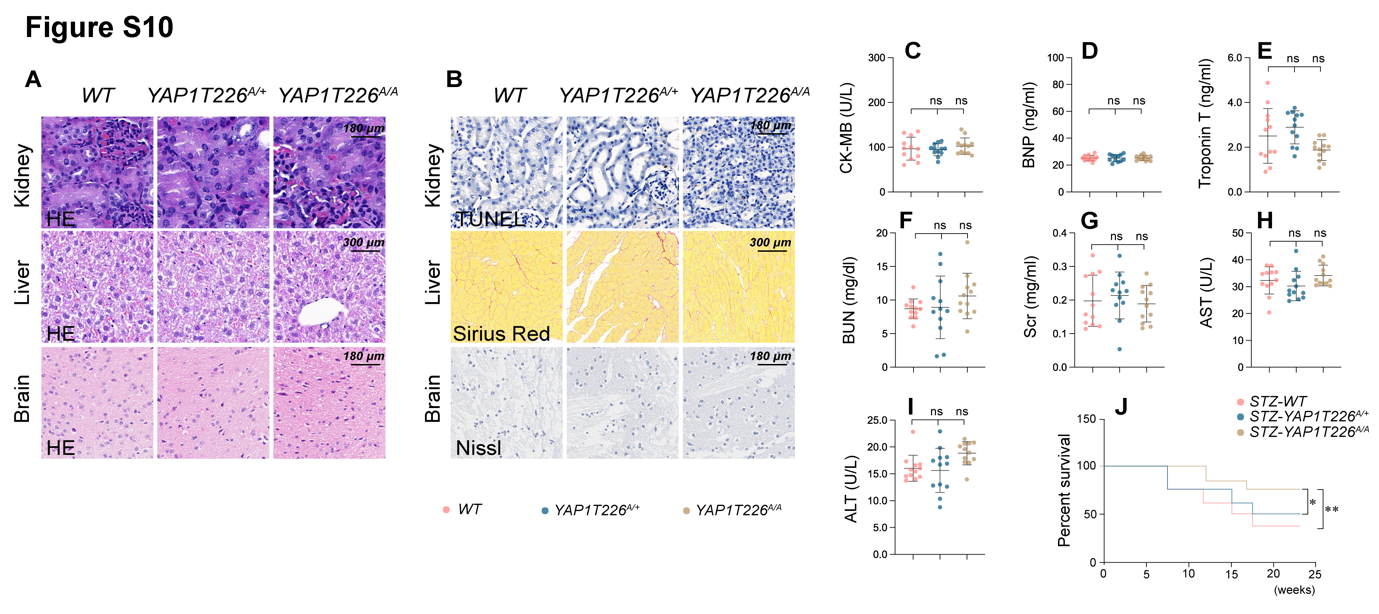


**Figure S11. NU7441 fails to provide additional cardioprotection in *YAP1^T226A^* mice under diabetic conditions.**

Heterozygous *YAP1T226^A/+^* mice, homozygous *YAP1T226^A/A^* mice, and wild-type (WT) mice (8-10 weeks of age) were injected intraperitoneally with streptozotocin (STZ, 50 mg/kg in 0.1 mol/L citrate buffer) for five consecutive days to induce diabetes. To inhibit the activity of DNA-PKcs, diabetic mice were intraperitoneally injected with NU7441 (2 mg/kg) for 24 weeks. Age- and sex-matched non-diabetic control mice were injected with an equal volume of PBS.

**(A-F)** Echocardiographic analysis of cardiac function.

**(G-I)** Quantitative PCR (qPCR) analysis of *Il-6,* *Tnfα*, *Mcp1*, and *Ptgs2* mRNA expression in heart tissues.

**(J)** ELISA kit was used to analyze the MMP9 activity.

**(K)** Representative histopathological images (Sirius Red staining) illustrating myocardial fibrosis in diabetic mice.

**(L)** Quantification of Sirius Red staining to assess myocardial fibrosis.

**(M-N)** qPCR was used to observe the transcription of ferroptosis-related genes including *Ptgs2* and *Slc1a5.*

Each group consisted of 4 animals or 4 independent cell culture experiments (n = 4). For each animal or independent cell culture experiment, measurements were repeated three times under the same experimental conditions. In each panel, dots represent individual measurements from animals or independent cell culture experiments. Bars represent group means, and error bars indicate ± standard error (SD). *p < 0.05; **p < 0.01; ***p < 0.001; ns, not significant.


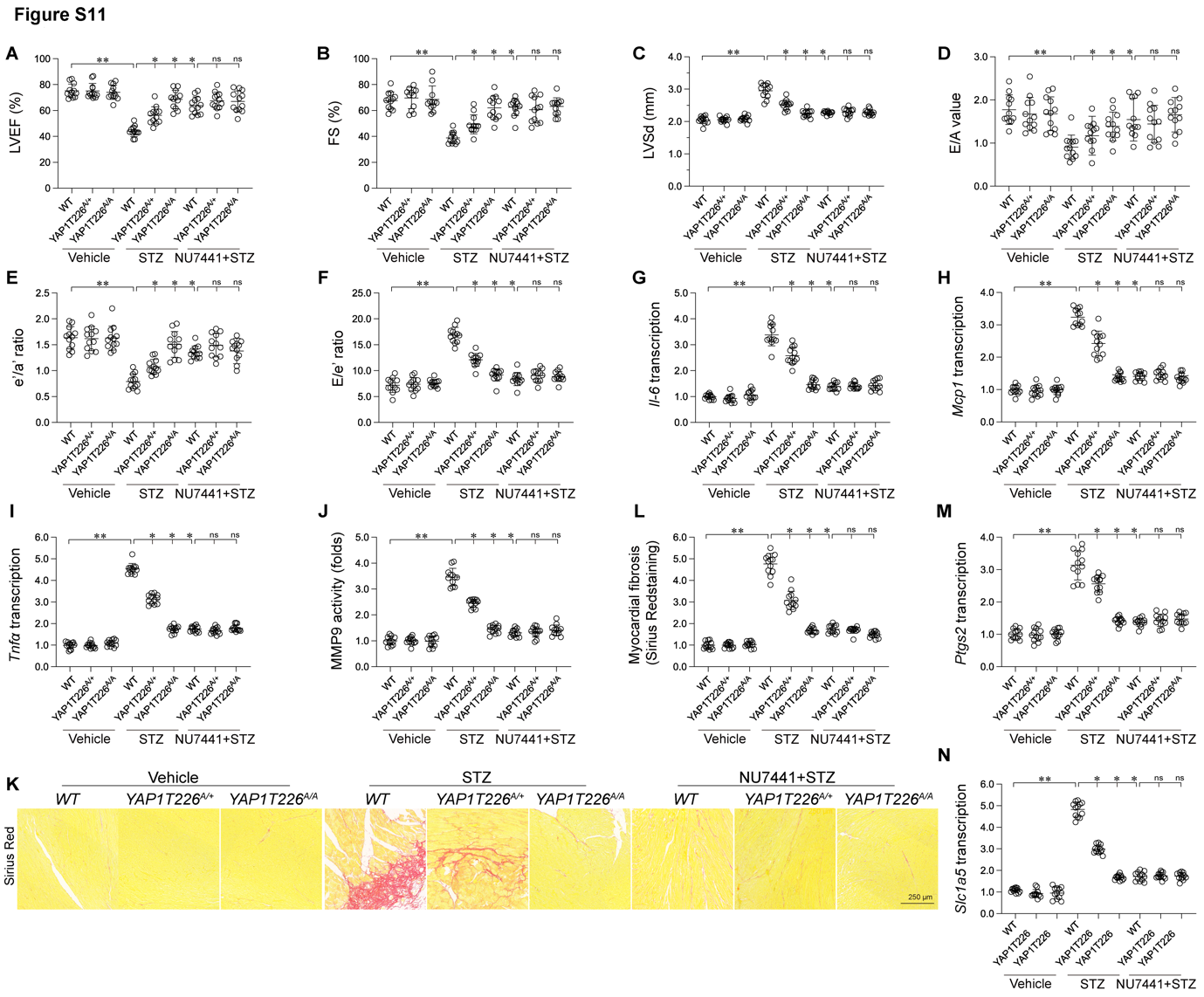

Supplement: Supplementary file 1 — Supporting Information [file ADVS-12-2412698-s001.docx]
